# A Possible Way for the Detection and Identification of Dangerous Substances in Ternary Mixtures Using THz Pulsed Spectroscopy

**DOI:** 10.3390/s19102365

**Published:** 2019-05-22

**Authors:** Vyacheslav A. Trofimov, Svetlana A. Varentsova

**Affiliations:** 1 School of Mechanical and Automotive Engineering, South China University of Technology, Guangzhou 510640, China; 2 Faculty of Computational Mathematics and Cybernetics, Lomonosov Moscow State University, Leninskiye Gory, Moscow 119991, Russia; svarentsova@gmail.com

**Keywords:** pulsed THz Time-Domain Spectroscopy, method of spectral dynamics analysis (SDA-method), integral correlation criteria, detection and identification of substance, ternary mixture, dangerous substances, reflected pulse, spectra of sub-pulses

## Abstract

We discuss an effective tool for the detection and identification of substances in ternary mixtures with similar spectral properties using a broadband reflected THz signal. Nowadays, this is an urgent problem; its effective solution is still far off. Two ternary mixtures of the explosives (RDX+TNT+HMX and RDX+TNT+PETN) were used as the examples for demonstration of the efficiency of the method proposed. The identification is based on the pulsed THz spectroscopy. We follow the spectral intensities together with the use of integral correlation criteria. They use the spectral line dynamics of the THz pulse reflected from the substance under investigation and that of the standard THz signal from database. In order to increase the accuracy and reliability of the identification, we analyze the partial non-overlapping time intervals, containing the main pulse of the reflected THz signal and the sequential sub-pulses. The main pulse is shown to contain information about high absorption frequencies (ν > 2.6 THz) of the mixture components. In the sub-pulses, the absorption frequencies of the components are detected in the range of low (ν < 2.6 THz) and high (ν > 2.6 THz) frequencies. The opportunity of distinguishing the mixtures with similar spectral properties is also shown.

## 1. Introduction

Currently, THz spectroscopy is widely used for the detection and identification of substances, especially for security and anti-terrorism applications. The photon energy of THz radiation is about one million times weaker than X-ray photon energy. Therefore, it does not cause harmful photoionization in biological tissues and is safe for both humans and animals. Moreover, the most common nonpolar substances are transparent to THz radiation, and they have pronounced spectral fingerprints in the range of the THz frequencies. That is why THz spectroscopy is actively used both security screening [1,2,3,4,5,6,7,8,9,10] and for numerous studies in the scientific field, as well as in applied fields including non-destructive testing [11,12,13], medical and pharmaceutical sciences [14,15], and food quality control [16].

One of the widely used techniques for the substance detection based on THz time-domain spectroscopy consists in comparison to the absorption frequencies of a substance under investigation with the known set of the absorption frequencies of the pure substances from a database. To develop such a database, the absorption frequencies of crystalline explosives (RDX, PETN, TNT, HMX, DNT, and Semtex) were investigated in the transmission mode in References [1,4,5]. The various kinds of pure illicit drugs (amphetamine group substances, heroin, acetyl codeine, morphine, and ketamine) as well as those hidden in mail envelopes were inspected and identified both in nitrogen and in the ambient air [2,3]. The spectra of pure explosives (RDX, HMX, PETN, and other substances) were measured in the reflection mode using the TDS system in [6,7]. There are many other papers dealing with developing such databases.

Below, we call this identification technique the standard THz-TDS method. Despite the obvious advantages of this safe and through-barrier method, it is still difficult to implement this method of substance detection in practice with high efficiency. One of the reasons follows from the fact that the absorption frequencies of explosives and other dangerous materials can be masked by non-opaque covering, clothing, and atmospheric water vapor absorption [8,9,10]. Scattering from the inhomogeneous surface of an explosive also results in spectrum distortion [17].

THz-TDS and THz spectroscopic imaging are widely used to study multi-component mixtures [18,19,20,21,22,23,24,25,26,27,28,29,30,31,32,33,34] such as chemical mixtures of neutral substances [18,19,20,21,22,23,24], explosives [25,26,27,28], illicit drugs [29], amino acids [30,31,32], and neurotransmitters metabolites in biomedicine [33]. THz-TDS is used for monitoring the components and content of PM2.5 particulates in the atmosphere [34] that is of great importance for pollution control and environmental protection. It is worth noting that, in most of these papers, in order to determine the components concentrations in the mixture, THz-TDS is used together with different chemometrics methods in the transmission mode.

Determining the concentration of pre-known substances in binary, ternary, or multicomponent mixtures is currently an urgent problem. In the nondestructive inspection of chemicals, including illegal drugs, explosives, and bioterrorism substances [18,19,20,21,22,23,24,25,26,27,28,29], one needs to quantitatively estimate mixture components and understand the role of each of them. For example, the nutritional value of protein in cereals [30,31,32] depends upon the contents of various amino acids in them. Therefore, determination of diversified essential amino acids in cereals is important for food processing and human nutrition. In biomedicine, in the real tissue cells, the proportions between the neurotransmitters and metabolites can reveal the status of tissue cells [33]. Seven substances that exist in brain glioma are chosen as the components of a mixture. They are identified on the base of the wavelet transform, baseline elimination, support vector regression, and loop iteration of samples. Thus, the quantitative analysis of such mixtures can be very useful for the early glioma diagnosis.

In [18,19] the components in chemical mixtures were quantitatively analyzed through THz spectroscopic imaging and component pattern analysis using known spectral data for pure chemicals. In Reference [20], four different chemicals were recognized by defining a recognition coefficient proportional to the height of absorption coefficients with respect to the spectral baseline. In Reference [21], several groups of the binary mixtures and a ternary mixture were investigated in the frequency range from 0.3 to 1.5 THz in transmission mode. A linear regression method was used to obtain relative contents in the mixtures. In References [22,26,27,28,30,31], the partial least-squares (PLS) algorithms, principal component analysis (PCA), and support vector machine (SVM) were used for quantitative analysis of the binary and ternary mixtures also in transmission mode. It should be reminded that PCA method allows reduction of the data dimension with minimal information loss; SVM is a well-known method used for the classification problems and it is considered as a special case of Tikhonov regularization.

The quantitative and qualitative analysis of calcium-based micro-fillers was performed in Reference [23] on the base of changing the spectral intensities at the absorption frequencies and their positions, and the appearance of additional features of the spectrum such as shoulders and small peaks. Self-modeling curve resolution (SMCR) technique was used in Reference [24] for the resolution of THz spectra of binary and ternary mixtures in the transmission mode. THz-TDS based on a combination of wavelet thresholding and wavelength selection was applied in Reference [25] for component analysis of the composition B-3 and pentolite in noisy and humid environments. A linear regression method was developed in Reference [29] to determine the relative contents of illicit drugs in the binary and ternary mixtures on the assumption that all components and their absorption frequencies are known. In Reference [32], the ternary amino acids in foxtail millet substrate were quantitatively analyzed. Utilizing three parameters derived from THz-TDS (absorption coefficient, extinction coefficient, and refractive index), the images were constructed, and the Chebyshev image moments were used to detect the mixture components. Then the quantitative models were obtained by stepwise regression. A back propagation (BP) neural network was applied in [34] for the qualitative and quantitative analysis of the mixtures. Recall that the back propagation method is a modification of the classical gradient descent method and in some cases, it is more efficient than the least squares method. However, BP neural network requires a large database to enhance its accuracy, which slows down the analysis.

Summarizing, it should be noted that THz-TDS together with different chemometrics techniques could be effective in many cases. Nevertheless, there are several restrictions for their application under real conditions. As usual, the main purpose of such investigations is to predict the concentration of the components in the complex samples. Moreover, these methods all require a large number of samples (the so-called training set) for reliable detection of the concentration of mixture components. However, in practice for the solution of the security problems, it is necessary to conduct the substance identification in real time. Thus, the processing of multiple measurements (using dozens or even hundreds of samples) reduces the performance of the security screening.

The second reason, which is more important, is related with the following fact. The THz signals are studied by means of THz-TDS and chemometrics methods in transmission mode. However, the security screening is typically carried out in the reflection mode. The spectra of the main pulses of reflected THz signals contain much less pronounced absorption frequencies than the transmitted ones. This complicates the identification by means of the standard THz-TDS, especially in the case of unknown mixture.

Nowadays there are also possibilities for substance detection with GHz sources, which thanks to a recent research on semiconductor superlattices (SSL) sources, and these sources may lead to competitive devices [35,36]. In Reference [35], the explosives TNT, NG, HMX, and RDX vapor composition are studied with the THz spectrometer based on coherent spontaneous radiation (CSR) effects in sub-THz frequency range. The prototype of a highly effective detector is demonstrated to be able to distinguish the absorption frequencies of the gases NO, N2O (vapor components of explosives TNT, NG, HMX, and RDX) in the atmosphere. However, these gases are also the natural components of the atmosphere and can be emitted by a large amount of organic substances. This may lead to the false alarms. However, this method is ineffective for the explosives hidden under a sealed cover, as in this case there is no evaporation, belonging to these materials and passed through the cover.

Unlike the methods mentioned above, we use only one sample as a standard sample to measure the standard signal in the transmission mode of the THz spectrometer and only one THz signal, reflected from the sample under analysis, for the detection of the mixture components. It is important that this reflected THz signal was measured in the long-time interval duration of about 180 ps. Thus, we explore not only the main reflected THz pulse, but also several sub-pulses. Their appearance will be discussed below. These sub-pulses contain information about absorption frequencies of the mixture components and can be used for the substance identification.

In the present paper, we continue developing of an effective tool for the detection of the components of two ternary mixtures of explosives (RDX+TNT+HMX and RDX+TNT+PETN) with similar spectral properties in THz frequency range under providing measurements in the reflection mode. We show that the main pulse spectrum of the measured THz signal does not have the pronounced absorption frequencies in the THz frequency range ν < 2.6 THz. Therefore, using of the standard THz-TDS method is inefficient for the detection and identification of the mixture components. In order to overcome the restrictions of this method, we propose to use the spectral dynamics analysis method (SDA-method) and several integral correlation criteria (ICC) simultaneously. In this method, the time-dependent spectral intensity (spectral line dynamics) at the chosen frequency for the signal passed through or reflected from a substance under analysis is compared with the corresponding spectral dynamics for the standard substances from a database.

It should be noted that the ICCs allow us not only to detect the presence of the dangerous components of the mixture but also to show the absence (or presence) of neutral substances within them. This is important for the security screening under real conditions because it reduces false alarms. As example, we consider the detection of paper and Si-based semiconductors in the ternary mixtures. Paper is a widespread packing material and any electronic device, such as a smartphone or notebook computer, contains semiconductor material.

Earlier in References [37,38,39], the SDA-method was used for the detection and identification of various substances in the transmission and reflection mode at a short (no more than 30 cm) distance between the receiver and an object under investigation. In References [40,41], we proposed using of the ICCs together with the SDA-method to achieve the detection with high probability. In References [42,43,44], the ICCs were applied for the substance detection and identification at long distance of about 3.5 meters under real conditions, which cause the highly noisy THz signal [45,46,47]. The essential limitations of the standard THz-TDS method also were demonstrated in [42,43,44,45,46,47].

In Reference [41], for the detection and identification of the ternary mixtures, mentioned above, we used one of the first types of the ICC, which takes into account the spectral brightness of the absorption frequencies of both the standard THz signal and the THz signal under investigation. The identification was carried out using the first sub-pulse only considering the main pulse reflected from the sample, as well as the remote part of the signal. One to three absorption frequencies of the mixture components were detected in the frequency range ν < 2.6 THz, which possess the high spectral amplitudes.

In the current paper, we apply, for this purpose, two ICCs simultaneously. The first ICC contains a term of the standard THz signal spectral brightness at the corresponding absorption frequency only, while the second one does not use the spectral brightness of the absorption frequencies of both signals at all. In order to increase the accuracy and reliability of the identification, both ICCs are applied in the partial non-overlapping time intervals. The first time interval contains the main pulse only and the second interval—the first sub-pulse, and the third—the following sub-pulses with lower spectral intensities. Moreover, we use the integral correlation between the THz signal, reflected from the sample under investigation, and the standard THz signal to find the time intervals, during which the signal under investigation is similar to the signals from database. Using these three criteria, compared to the application of a single criterion in a single time interval, increases the probability and reliability of the substance detection and identification. In addition, we show that the ICCs allow the detection of not only the low absorption frequencies belonging to the mixture components and possessing the high spectral brightness, but also the higher absorption frequencies possessing lower spectral brightness.

Using the ICCs, it is possible to show the absence of the substance of interest in the sample under investigation. For this aim, it is sufficient to analyze the long-term reflected THz signal and the second type of the ICC, together with the analysis of the reference spectrum.

The measurements of THz pulses reflected from the mixtures RDX+TNT+HMX and RDX+TNT+PETN were made in the Military University of Technology (Warsaw, Poland) and the investigation of them are described in Reference [39], where the absorption frequencies of all components were found analyzing the first sub-pulse only.

The transmitted THz signals through RDX, HMX, PETM, and TNT were measured in the Center for Terahertz Research, Rensselaer Polytechnic Institute (NY, USA). They were also used as the standard signals in Reference [37]. The THz signals transmitted through the sample with Si-based semiconductor n-Si and paper were registered at the Capital Normal University (Beijing, China) and the South China Normal University (Guangzhou, China), respectively. In References [43,48], absorption frequencies of these signals were detected by the SDA-method and the first type of the ICC.

## 2. Identification of Ternary Mixtures of Explosives in Reflection Mode

In Section 2, we discuss the absorption frequencies of two mixtures RDX+TNT+HMX and RDX+TNT+PETN and apply the SDA-method together with different types of the ICCs for the detection and identification of the mixture components. For brevity, we below call these signals as RTH signal for the mixture RDX+TNT+HMX and RTP signal for the mixture RDX+TNT+PETN. The absorption frequencies as well as the detection and identification of the substance components are provided using different parts of the THz signal measured in the reflection mode. In our opinion, these tools allow us to increase the detection probability. We stress that our method is reference-free. However, we compute the reference spectrum in order to exclude the false absorption frequencies, induced by noise, in the spectra of the RTH and RTP THz signals, but we do not use the reference to compute reflectance and refractive index of the substance under consideration. However, the reference may be used as an additional characteristic for increasing of the detection probability.

The samples were prepared as follows. The components were ground into fine powder to decrease the particle size and to avoid scattering loss. Then, they were mixed with a few percent of a pure polyethylene and pressed into the pellets with an equal ratio of individual components. The weight of each pellet is 600 mg and diameter is 13 mm. The reflected THz signals were measured using Teraview TPS 3000 spectrometer in the standard stand-off reflection configuration. The main parameters of the system are: Spectral range 0.06–3.6 THz. The signal-to-noise is better than 4000:1; the dynamic range optical density is higher than 3 in the range 2 cm^−1^ to 100 cm^−1^. The spectral resolution is 0.06 THz and the rapid scan mode is 30 scans/second. The samples were placed nearly perpendicular to the beam—with 6° incident angle for 300 mm distance in the cuvette. Measurements were made in the compartment purged with nitrogen to avoid the effect of water vapor absorptions in ambient air. All measured THz signals have duration of about 180 ps. A gold mirror reflecting more than 99% of incident radiation was used to obtain the reference. A detailed description of the physical experiment with mixtures RTH and RTP is given in Reference [39].

Measurements of the THz pulses transmitted through the explosives RDX, HMX, PETN and TNT were made also using Teraview TPS 3000 spectrometer in the standard configuration. The distance between a sample and the receiver did not exceed 30 cm. The measurement procedure is described in detail in [4], but in our case, the RDX, HMX, PETN, and TNT signals were measured in the ambient air [37]. Typically, “pure” standard THz signals, measured in a dry atmosphere, are used for the substance detection and identification. However, we used THz signals measured under real conditions for two reasons. First, we immediately eliminate the water absorption frequencies from our consideration. Second, we demonstrate in such a way that the feasibility and applicability of developed tools even at using the standard signals disturbed by water vapor.

A Ti:sapphire laser (Spectra-Physics Mai Tai Inc.) was applied to generate and detect the THz pulse transmitted through semiconductor n-Si with a duration of 100 fs and a central wavelength of 800 nm at a repetition rate of 1 kHz, with noise less than 0.15% [49,50,51]. The path with THz radiation was purged by nitrogen to prevent absorption by atmospheric water vapor. The n-Si sample was 0.5 mm thickness.

Measurements of the THz signal transmitted through the paper sheet, were carried out at room temperature in the absence of water vapor influence by using a Ti:sapphire femtosecond oscillator (Mira 900, Coherent) pumped with a solid-state laser (Verdi-5, Coherent). The details of the experiment can be found in Reference [52].

### 2.1. Temporal Structure of the Reflected Signals RTH, RTP and Spectra of Their Main Pulses

**Figure 1:**
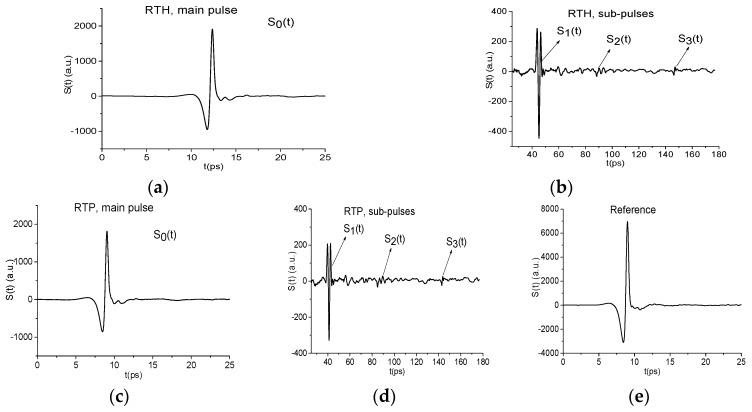
Reflected THz RTH (**a**,**b**) and RTP (**c**,**d**) signals measured in the time intervals t = [0, 25] ps (**a**,**c**) and [25, 180] ps (**b**,**d**). Reference is measured in the time interval t = [0, 25] ps (**e**).

In Figure 1 the THz signals reflected from the pellets with mixtures RDX+TNT+HMX (a), (b) and RDX+TNT+PETN (c), (d) are presented in two time intervals t = [0, 25] ps (a), (c) (main pulse) and t = [25, 180] ps (sub-pulses, following the main pulse). We will use each of these signal parts for the detection and identification of substance.

The structure of the RTH and RTP signals is clearly seen in these figures–they contain the pronounced main pulse $S_{0}(t)$ (a), (c) reflected from the sample, and several sub-pulses $S_{1}(t)$, $S_{2}(t)$, $S_{3}(t)$ (b), (d) caused by multiple reflection from tablet boundaries. In (e), the reference is given, which is common for both RTH and RTP signals. Their amplitudes are about 3.88 times less than the reference amplitude due to transmission of the THz signals through the sample. Obviously, a part of the signal energy is absorbed by the sample. Below, we discuss briefly the absorption frequencies belonging to the main pulses of the reflected RTH and RTP signals, which were investigated earlier in References [39,41]. They are necessary for the detection of substance using the ICCs.

Probably, it is necessary to discuss an appearance of these sub-pulses in detail. First of all, we are reminded that several processes simultaneously occur in the tablet after penetration of the THz pulse inside it. The pulse undergoes multiple reflections. Obviously, this process depends on the relationship between the thickness of the tablet and the spatial length of the pulse. The pulse may occupy only a part of the tablet or its spatial length will be greater than the tablet thickness. In the first case, we will see a sequence of the pulses leaving the tablet during equal time intervals. In the opposite case, we will see continuous emission from the tablet. The second physical reason, which influences on the sequence of the pulses, is a dispersion of a medium. Due to the THz pulse is broadband, and the spectral harmonics propagate with different velocities, the emission from the tablet will be with changing delays and emitted pulses do not have sharp duration: they will overlap each other. The third characteristic feature of the THz emission results from the pulse interaction with a medium inside the tablet. Due to a broadband spectrum of the THz pulse, an excitation of various energy levels of molecules occurs. Then there is a relaxation of these energy levels through the radiative or non-radiative energy level transitions. Non-radiative transitions are responsible for the absorption frequencies of a medium. Radiative energy level transitions lead to appearance of new spectral harmonics which may have frequency either less than the frequency corresponding to the frequency of excited energy level transition or greater than this frequency due to the cascade mechanism of the energy level excitation (we described this mechanism in [53]). Therefore, from an analysis of the pulse spectrum we will see the emission frequencies and the absorption frequencies caused by appearance of the emission of a medium at some frequencies. All these processes (excitation and emission) occur with different relaxation time. Therefore, a medium response on the emission frequencies as well as on certain absorption frequencies will be at different time. That is why we see a non-equal time delay between the pulses in their sequence. With time, we see continuous response of a medium. However, this circumstance does not restrict the proposed method applicability because using the correlation function-based math tool it is possible to find a time interval, which corresponds to the processes of the emission or absorption at the radiative energy level transitions. We demonstrated this fact during an analysis of a noisy signal when the noise amplitude is greater than the amplitude of the useful signal [45].

One more important question relates to an influence of the tablet thickness on the response of a medium under THz pulse propagation. In our opinion, we have to take into account at least two physical mechanisms when the tablet thickness will be decreased. The first of them relates to the reflection of the THz pulse (and therefore, to the emission discussed above). At decreasing of the tablet thickness, we will observe the reflected THz pulse at higher frequencies because a radiation wavelength, at which obstacle (it means the tablet) will act on the radiation, must be decreased. The second one relates to the scattering of THz radiation with wavelength, which is greater than the tablet thickness. The scattering intensity is proportional to the ratio of the tablet thickness to the wavelength in power 4. We will observe this scattering also. Of course, these two processes will occur simultaneously together with a presence of the absorption and emission of the THz radiation. Consequently, we will see a complicated picture of the THz pulse interaction with such a tablet. Nevertheless, we will observe the reflected THz pulse at high frequencies and the sub-pulses (or only one sub-pulse with long duration), which follow the main pulse. The reflected sub-pulse will contain information about the absorption and emission frequencies in any case, but spectral power density at these frequencies as well as the frequency range, at which this response will be observed, will shift.

**Figure 2:**
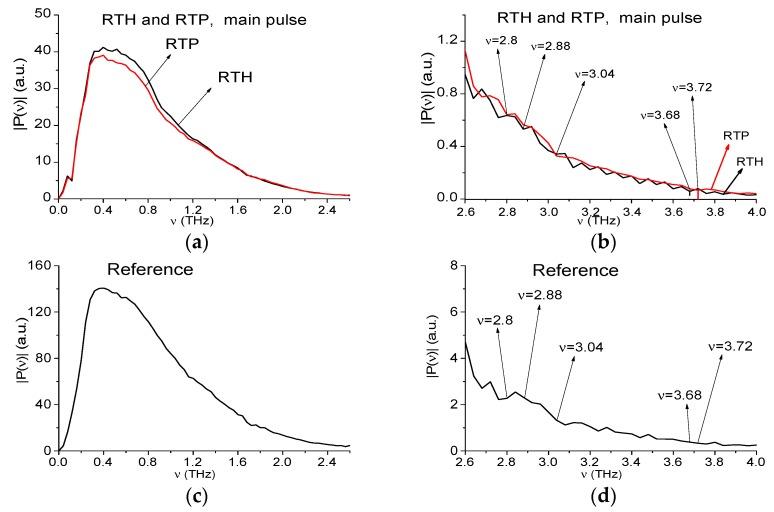
Spectra of the RTH, RTP main pulses (**a,b**) and reference spectrum (**c,d**) depicted in the frequency ranges ν = [0, 2.6] THz (**a,c**) and [2.6, 4.0] THz (**b,d**). The corresponding spectral resolution is equal to Δ*ν* = 0.04 THz.

As a rule, the main pulse of the reflected THz signal is the object of the analysis when using the standard THz-TDS. In Figure 2 the RTH and RTP main pulse spectra as well as reference spectrum are shown in the frequency ranges ν = [0, 2.6] THz (a), (c), [2.5, 4.0] THz (b), (d), respectively, at the frequency resolution Δ*ν* = 0.04 THz. We have divided the frequency range conventionally into two parts: ν ≤ 2.6 THz (low frequency range) and ν > 2.6 THz (high frequency range), because the spectral intensities of the measured THz signals in these ranges are essentially different. Let us note that decreasing of the RTH and RTP spectral intensities (Figure 2a) compared to the reference ones (Figure 2c) is in agreement with the decreasing of the amplitudes of these signals (see Figure 1).

One can see that both spectra in (a) do not contain any pronounced minima in the low frequency range *ν* = [0.0, 2.6] THz, and the reference spectrum (c) does not contain them either. Thus, reflectance R(ν) also does not demonstrate the absorption maxima (not shown). Reflectance R(ν) is computed as a ratio

$$R\left(\nu \right)=\left|P\left(\nu \right)\right|/\left|P_{REF}\left(\nu \right)\right|,$$

where |P(ν)|, $\left|P_{REF}(\nu )\right|$ are the amplitudes modulus of the RTH main pulse and reference spectra, respectively. It should be stressed that in Reference [6,7] the well-known RDX absorption frequency *ν* = 0.82 THz is also not visible in the reflectance.

About a chosen frequency resolution, it is necessary to note that earlier in Reference [54] we showed that increasing of the spectral resolution for the noised signal leads to observing of the small-scale distortion in the signal and its spectrum, which are caused by packing material, or the material structure. Decreasing the spectral resolution results in excluding of the false absorption frequencies as well as in detecting of the frequencies caused by water vapor absorption. Therefore, there is an optimal spectral resolution, which depends on a bandwidth of the absorption line of the substance. For example, taking into account the half-width of the spectral line for the RDX absorption frequency ν = 0.82 THz, we get that the optimal spectral resolution is the value Δ*ν* = 0.04 THz. Hence, in the present paper, the RTH and RTP main pulse spectra are computed with the spectral resolution Δ*ν* = 0.04 THz.

Before providing the spectrum analysis, we are reminded that RDX possesses the following absorption frequencies in the frequency range *ν* = [0, 4.0] THz: ν = 0.82, 1.05, 1.36, 1.54, 1.95, 2.19, and 3.08 THz. Those of HMX are ν = 1.78, 2.51, 2.82 THz, those of PETN- ν = 2.0, 2.16, 2.84 THz, and those of TNT are ν = 1.62, 2.20, 3.69 THz [4,5]. In Figure 2b the RTH and RTP main pulse spectra are depicted in the high frequency range *ν* = [2.6, 4.0] THz. One can see the spectrum minimum at the frequency ν = 3.04 THz for both signals. This minimum is close to the RDX absorption frequency ν = 3.0 THz. We see also a minimum in the RTH signal spectrum at the frequency ν = 2.88 THz close to the HMX absorption frequency ν = 2.84 THz, and a minimum in the RTP signal spectrum at the frequency ν = 2.8 THz close to the PETN absorption frequency ν = 2.82 THz. Moreover, the minimum at the frequency ν = 3.68 THz is observed in the RTH signal spectrum, and at the frequency ν = 3.72 THz–in the RTP signal spectrum. These minima are close the TNT absorption frequency ν = 3.69 THz [5]. At the same time, the spectral minima at these frequencies are absent in the reference spectrum (d). That means that in the RTH and RTP signals spectra (b) minima mentioned above are not caused by environmental influence or background noise and they may be the absorption frequencies of the RTH and RTP mixtures components. However, this is not sufficient for the detection and identification of these mixtures by means of the standard THz-TDS method. The reason is the following. It is obvious that the RTH and RTP mixtures do not contain neutral substances except pure polyethylene, which is transparent to THz radiation [55]. However, the spectra (Figure 2b) contains several minima (*ν* = 2.6, 2.88, 3.04, 3.68 THz (RTH)) and *ν* = 2.64, 2.8, 3.04, 3.72 THz (RTP)), which may correspond to the paper sheet and n-Si semiconductor absorption frequencies. Therefore, we have to be sure that the absorption frequencies observed in RTH and RTP spectra do not belong to these materials. In this connection, we need to be able to differentiate between these neutral substances and dangerous ones despite the fact that they possess the same absorption frequencies.

**Figure 3:**
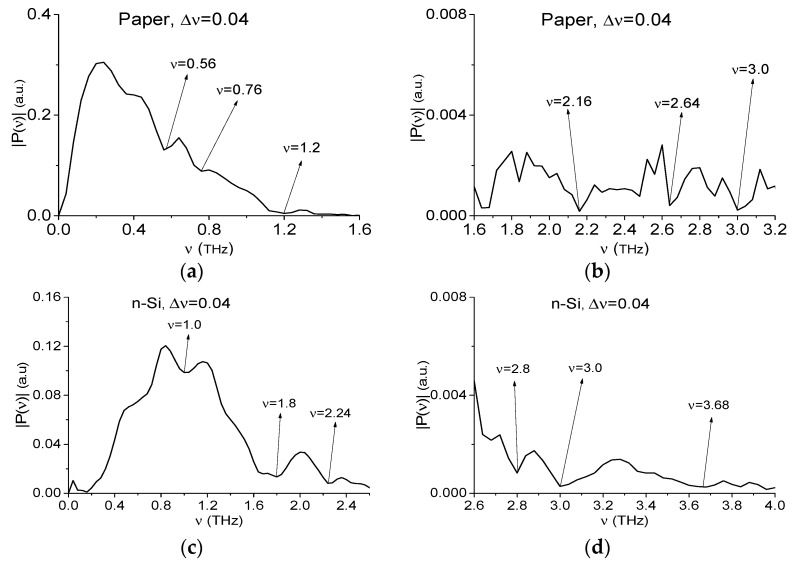
Spectra of the THz signals, transmitted through the paper (**a,b**) or n-Si (**c,d**), depicted in the frequency ranges ν = [0, 1.6] THz (**a**), [1.6, 3.2] THz (b), ν = [0, 2.6] THz (**c**) and [2.6, 4.0] THz (**d**). The corresponding spectral resolution Δ*ν* = 0.04 THz.

We will call the THz signals transmitted through these substances, as the paper signal and n-Si signal respectively, for brevity. In Figure 3 their spectra are depicted. In (a), (b) one can see minima at the frequencies *ν* = 0.56, 0.76, 1.2, 1.64, 2.16, 2.64, and 3.0 THz and these values are in a good agreement with the absorption frequencies of the paper reported in Reference [56] in the frequency range ν > 2.0 THz: ν = 2.1, 2.5, 3.1 THz. In (c), (d) the minima of the n-Si spectrum are achieved at the frequencies: ν = 1.0, 1.8, 2.24, 2.8, 3.0, and 3.64 THz. As a result, in the RTH, RTP spectra Figure 2b the frequencies ν = 2.88, 3.04, 3.68 THz (RTH) and ν = 2.8, 3.04, 3.72 THz (RTP) can be identified as those of the n-Si spectrum (Figure 3d). At the same time, the spectra minima at the frequencies ν = 2.6 THz (RTH), 2.64 THz (RTP) are close to those of the paper spectrum (Figure 3b). That is why we said above that the correspondence of the RTH and RTP spectral minima to the frequencies from database is insufficient for claiming that the substance is the dangerous one. Therefore, one can detect the neutral substances in the mixtures instead of the dangerous substance Figure 2b (or vice versa). This means a low efficiency of the standard THz-TDS method for the detection and identification of substances at using only the main pulse of the reflected THz signal even under laboratory conditions. One of such methods, which may be effective for the detection and identification of the mixture components, is the SDA-method at using the ICCs.

### 2.2. Brief Overview of Integral Correlation Criteria and the SDA-Method

In this Section, we remind the main idea of the SDA-method and ICCs. Note that the computation of the spectral intensity dynamics is described in a few of our previous papers, for example, in References [44,45]. For quantitative estimation of a presence of a substance of interest in a sample, we proposed in Reference [42] using of the integral correlation between the spectral intensity dynamics of the THz signal under investigation S(t) (reflected from or transmitted through the substance) and that of the standard transmitted signal s(t) belonging to the database.

Let $p_{\nu_{1}}=\left\{\left|p_{\nu_{1}}(t_{m})\right|\right\}$, $m=0,\ldots ,M_{1}-1$, denote the set of spectral intensities absolute values for the standard signal s(t) at the chosen frequency $\nu_{1}$ and $P_{\nu_{2}}=\left\{\left|P_{\nu_{2}}(t_{m})\right|\right\}$, $m=0,\ldots ,M_{2}-1$, denote the corresponding set for the signal S(t) at the frequency $\nu_{2}$. $P_{\nu_{2}}{}^{\left(n\right)}=\left\{\left|P_{\nu_{2}}^{\left(n\right)}(t_{n+m})\right|\right\}$ denotes its part with $M_{1}$ components that begins at time moment $t_{n}$. Here $M_{1}$ and $M_{2}$ are the numbers of time moments in the dynamics, which are computed in the whole time intervals. They depend on the construction parameters - the window length T and its shift Δ along the signal under investigation because we use the Fourier-Gabor method. In the current paper, as in References [43,44,45,46,47,48], these parameters were chosen as follows: The window length T = 2.8 ps and its shift Δ = 0.2 ps.

In order to compute the correlation coefficient between the sets $p_{\nu_{1}}$ and $P_{\nu_{2}}$ in each of the time moments $t_{n}$ we use the well-known expression:

(1) $$c_{p,P}(t_{n})=\sum_{m=0}^{M_{1}-1}\left(|p_{\nu_{1}}(t_{m})|-\bar{p_{\nu_{1}}})\cdot (|P_{\nu_{2}}(t_{m+n})|-\bar{P_{\nu_{2}}}\right)/||p_{{}_{\nu_{1}}}-\bar{p_{\nu_{1}}}||\cdot ||P_{\nu_{2}}^{\left(n\right)}-\bar{P_{\nu_{2}}}||$$

where $\bar{p_{\nu_{1}}}=\frac{1}{M_{1}}\sum_{m=0}^{M_{1}-1}\left|p_{\nu_{1}}(t_{m})\right|$, $\bar{P_{\nu_{2}}}=\frac{1}{M_{1}}\sum_{m=0}^{M_{1}-1}\left|P_{\nu_{2}}(t_{m+n})\right|$ characterizes the non-zero constant average values in the sets $p_{\nu_{1}}$ and $P_{\nu_{2}}$. Their presence increases the correlation coefficient $c_{p,P}(t_{n})$ (1) and decreases the accuracy and reliability of the identification. Therefore, the normalization of the averaged value of the signal to zero-value is necessary to eliminate the influence of the constant values, containing in the spectral dynamics $p_{\nu_{1}}$ and $P_{\nu_{2}}$, on the correlation coefficient $c_{p,P}(t_{n})$.

It should be stressed that the local correlation coefficient (i.e., computed at a single time moment) cannot be used to show the presence or absence of the standard substance absorption frequencies in the THz signal under investigation because of their strong oscillations at different time moments, which are caused by the medium response oscillations due to noise presence, for example. Summing the correlation coefficients over the time interval allows us to “accumulate” the useful signal and to suppress the correlation coefficient fluctuations at different time moments. As is well known, this procedure is widely used for processing of noisy signals.

For the substance detection and identification, we use some ICCs, which are based on the correlation coefficient (1):

(2) $$C_{p,P}(t_{n})=\sum_{k=0}^{n}\left|c_{p,P}(t_{k})\right|,\;n=0,\ldots ,M_{2}-M_{1}$$

- time-dependent integral correlation of the spectral dynamics,

(3) $$CW_{p,P}(t_{n})=\sum_{k=0}^{n}\left|c_{p,P}(t_{k})\right|w_{1}w_{2},\;n=0,\ldots ,M_{2}-M_{1}$$

- time-dependent integral correlation of the spectral dynamics with taking into account the spectral intensity $\left|p(\nu_{1})\right|$, $\left|P(\nu_{2})\right|$ at each of the frequencies $\nu_{1}$ and $\nu_{2}$ for both signals. Here $w_{1}=1/|p(\nu_{1})|$, $w_{2}=1/|P(\nu_{2})|$ are the weight coefficients.

In particular, if we choose the weight coefficients in the following way: $w_{1}=1/|p(\nu_{1})|$, $w_{2}=1$ then we follow the criterion:

(4) $$CW1_{p,P}(t_{n})=\sum_{k=0}^{n}\left|c_{p,P}(t_{k})\right|w_{1},\;n=0,\ldots ,M_{2}-M_{1}$$

- time-dependent integral correlation of the spectral dynamics with taking into account the spectral brightness of the signal belonging to database. In comparison with the ICC $CW_{p,P}(t_{n})$ (3), the ICC $CW1_{p,P}(t_{n})$ (4) uses only the spectral brightness of the standard signal s(t), not the signal under investigation S(t). This allows us to be less dependent on random fluctuations of the measured signal and to rely upon the spectral characteristics of the standard signal.

Below, we use the ICCs for the detection of a desired substance in a mixture. For this purpose, we apply both ICCs $CW1_{p,P}$ (4) and $C_{p,P}$ (2) simultaneously to the THz signal under investigation during the partial non-overlapping time intervals. The first time-interval contains the main pulse only, the second one - the first sub-pulse, and the third interval - the following sub-pulses with lower amplitude. As an additional criterion of the detection, we use the integral correlation $C_{s,S}$ (6) (see below) between the measured THz signal S(t) reflected from the sample and the standard THz signal s(t) from the database. This allows us to find the time intervals, in which the response of a medium contains a signal similar to the signal from database. Therefore, the spectral analysis for the detection of this dangerous substance must be made only during these time intervals. It increases both the detection probability and a detection system rate.

The ICCs can also be used to show the absence of the substance of interest in the mixture using reflected THz signal. If there is a spectrum minimum in the main pulse or in the sub-pulses spectra, which coincides with some absorption frequency of this substance, then using of ICC $C_{p,P}$ together with the analysis of the reference spectrum is sufficient to show that this minimum does not belong to the absorption frequencies of the substance. In the transmission mode this result was shown for a noisy THz signal in Reference [45].

For estimation of a presence or absence of the substance of interest in the sample under investigation, in References [44,45], we introduced the following rules at using the ICC. The frequency ν is judged to be detected in the spectrum of the signal under investigation, if the values of the corresponding ICC computed for the pair $\left(\nu ,\nu_{1}\right)$, are greater than the values of this ICC computed for all other frequency pairs $\left(\nu^{*},\nu_{1}\right)$ in the frequency detection range (FDR). Here the frequency $\nu_{1}$ is the absorption frequency of a standard signal spectrum; the frequency $\nu^{*}$ belongs to the FDR. Vice versa, the frequency ν is not detected if there is at least a single frequency pair $\left(\nu^{*},\nu_{1}\right)$, whose ICC values are greater than the values of the ICC computed for the pair $\left(\nu ,\nu_{1}\right)$ in this FDR. As boundaries of the FDR, the spectrum maxima closest to the frequency under analysis were used. However, at analysis of the mixture, the boundaries of FDR must be defined by two ICC $CW1_{p,P}$ and $C_{p,P}$ simultaneously (we call this FDR as a minimal one).

It should be noted that under real conditions the thickness of the samples is not known as a rule. It is also obvious that the measurement conditions of the standard pure substance from database and the analyzed sample also are different. However, the integral correlation allows us to find similarity in the shape of the signals and dynamics of spectral lines, despite the difference in the thickness of the samples and different experimental conditions. That is why the ICCs can be an effective tool for the detection of the components of the mixtures.

### 2.3. Time-Dependent Analysis of the Similarity between the RTH Signal and the Standard THz Signals

In this section, we examine the similarity between the RTH signal and the standard signals from database corresponding to various dangerous and neutral substances. For this purpose, we compute the correlation coefficients between the reflected RTH signal (and RTP signal) and the standard THz signals. In order to do this, we move the standard signal $s(t)=\{s(t_{m})\}$, $m=0,\ldots ,N_{1}-1$ along the reflected signal $S(t)=\left\{S(t_{m})\right\}$, $m=0,\ldots ,N_{2}-1$. Here $N_{1}$ and $N_{2}$ are the numbers of time moments in these signals. Then, we compute the time-dependent correlation coefficient $c_{s,S}(t_{n})$ like (1), where instead of sets $p_{\nu }$ and $P_{\nu }$, we use the sets $s(t_{m})$ and $S(t_{m+n}):$

(5) $$c_{s,S}(t_{n})=\frac{\sum_{m=0}^{N_{1}-1}\left(s_{AV}(t_{m})\cdot S_{AV}(t_{m+n})\right)}{\left||s_{AV}(t_{m})||\cdot ||S_{AV}(t_{m+n})|\right|},n=0,\ldots ,N_{2}-N_{1},$$

where $s_{AV}(t_{m})=s(t_{m})-\bar{s},S_{AV}(t_{m+n})=S(t_{m+n})-\bar{S},$ $\bar{s}=\frac{1}{N_{1}}\sum_{m=0}^{N_{1}-1}s(t_{m})$, $\bar{S}=\frac{1}{N_{1}}\sum_{m=0}^{N_{1}-1}\left|(t_{m+n}\right)$.

As above, we substrate $\bar{s}$, $\bar{S}$ from the corresponding signals to avoid an influence of constants components of the signals on their correlation.

**Figure 4:**
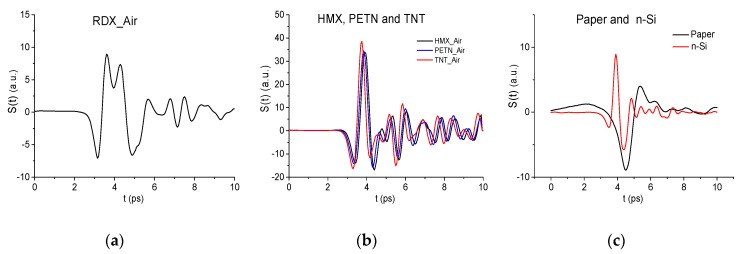
Standard THz signals RDX_Air (**a**)**,** HMX_Air, PETN_Air, TNT_Air (**b**)**,** and paper, n-Si (**c**).

As the standard signals, we use the transmitted THz signals RDX_Air, HMX_Air, PETN_Air, TNT_Air [40], and the main pulses of the THz signals transmitted through the paper and semiconductor n-Si [43]. To exclude an influence of different signal duration on the signal correlation, all standard signals durations are chosen to be equal to 10 ps and their maxima were located in the time interval t = [3,6] ps, they are shown in Figure 4a–c. The computation of the correlation coefficients $c_{s,S}(t_{n})$ starts at the point t = 0 ps. We see, in Figure 4b, the high similarity between the signals transmitted through HMX, PETN, and TNT; this is necessary to keep in mind at the analysis of the substance identification results.

**Figure 5:**
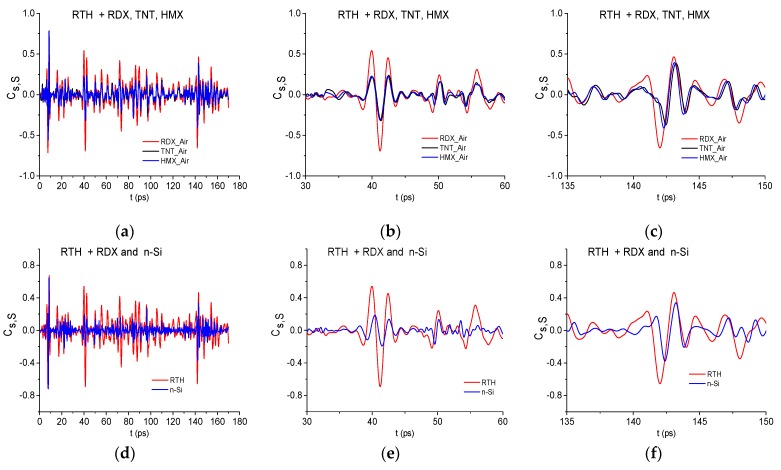
Time-dependent correlation coefficients $c_{s,S}$ between the RTH signal and the standard signals RDX_Air, TNT_Air, HMX_Air, (**a–c**)**;** between the RTH and RDX_Air, n-Si signals (**d–f**), depicted in the time intervals t = [0,180] ps (**a,d),** [30, 60] ps (**b,e**), [135, 150] (**c,f**).

Figure 5 shows time-dependent correlation coefficients $c_{s,S}$ between the long reflected RTH signal and the standard signals RDX_Air, TNT_Air, HMX_Air (a)–(c), n-Si (d)–(f) during various time intervals. One can see in (a) and (d) that the pronounced maxima of the correlation coefficients achieved high values in the time interval *t* = [20,120] ps, which does not contain the RTH main pulse. Therefore, we depict two time-intervals separately: t= [30, 60] ps (b), (e) and [135, 150] ps containing the first and the third sub-pulses of the RTH signal, respectively. The second sub-pulse has an inverted absolute phase with respect to the standard signal and is less visible. Therefore, we do not show the correlation coefficients for this sub-pulse here. We see that the correlation coefficient $c_{s,S}$ maxima obtained for the pair of signals RTH and RDX_Air are greater than the corresponding values for the pairs of signals RTH and TNT_Air, RTH and HMX_Air (b), (c), RTH and n-Si (e), (f) in these time intervals. So, we obtain the following values: $c_{RDX,RTH}$ = 0.542, $c_{TNT,RTH}$ = 0.22, $c_{HMX,RTH}$ = 0.23 (b); $c_{RDX,RTH}$ = 0.464, $c_{TNT,RTH}$ = 0.38, $c_{HMX,RTH}$ = 0.39 (c). For the n-Si standard signal and RTH signal we get the correlation coefficients: $c_{n-Si,RTH}$ = 0.186 (e), $c_{n-Si,RTH}$ = 0.34 (f). Since the standard signals HMX_Air and TNT_Air are similar to each other (Figure 4b), then we obtain a proximity of the correlation coefficients $c_{TNT,RTH}$ and $c_{HMX,RTH}$ maximal values in (b), (c). The same is valid for the PETN_Air signal: $c_{PETN,RTH}$ = 0.24 in the time interval t = [30, 60] ps and $c_{PETN,RTH}$ = 0.4 in the interval t = [135, 150] ps (not shown).

Thus, we see that the correlation coefficient $c_{s,S}$ possesses a big value changing over the time interval under consideration. Therefore, we introduce the following integral characteristic for two signals:

(6) $$C_{s,S}(t_{n})=\sum_{m=0}^{n}\left|c_{s,S}(t_{m})\right|,\;n=0,\ldots ,M_{2}-M_{1}.$$

**Figure 6:**
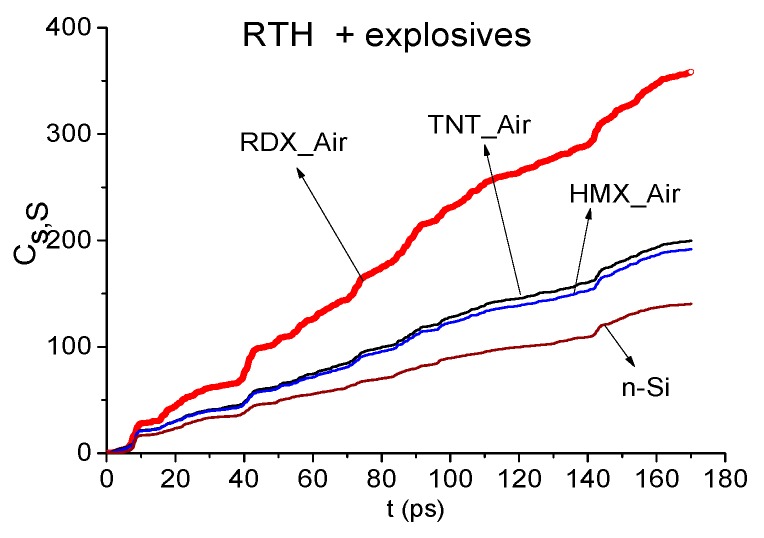
Time-dependent ICC $C_{s,S}$ computed for the RTH signal and the standard signals RDX_Air, TNT_Air, HMX_Air and n-Si in the time interval [0, 180] ps.

It allows us to increase a probability of the identification of substance. In Figure 6 the ICC $C_{s,S}$ is shown for the RTH signal and the standard signals RDX_Air, HMX_Air, TNT_Air and n-Si in the time interval t = [0, 170] ps. This figure demonstrates that the integral correlation between the RTH signal and the standard RDX_Air signal is essentially greater than that one computed for other chosen standard signals belonging to explosives or semiconductor n-Si. In the time intervals t = [30, 70] ps, [70, 110] ps, and [120, 160] ps containing the sub-pulses, the integral correlation between RTH and RDX_Air signals is also greater than for other standard signals. We note that the same results occur for the RTP signal.

**Figure 7:**
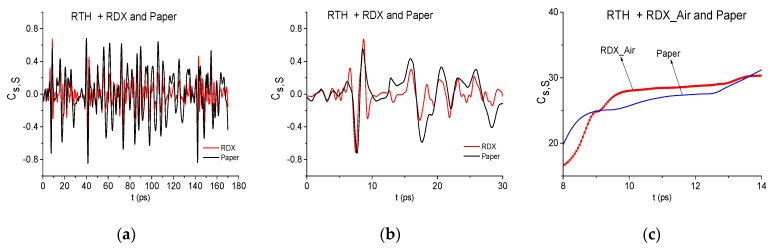
Correlation coefficients $c_{s,S}$ between the RTH signal and RDX_Air standard signal (red line) as well as between RTH signal and paper standard signal (black line) computed in the time intervals t = [0,180] ps (**a**) and [0,30] ps (**b**). ICC $C_{s,S}$ computed between these signals in the time interval t = [8,14] ps (**c**).

However, it is still not enough for the reliable detection of the substance RDX in these mixtures. Indeed, the correlation coefficient $c_{s,S}$ between the RTH signal and the standard RDX_Air signal (Figure 7a) demonstrates higher degree of their similarity, in comparison with the correlation between the RTH signal and the standard paper signal, only in the short time interval t = [8, 14] ps (Figure 7b). The evolution of the ICC $C_{s,S}$ computed in this time interval confirms this fact (Figure 7c). Therefore, we need to apply other criteria for the detection and identification of substance.

### 2.4. Absorption Frequencies of the Standard THz Signals

**Figure 8:**
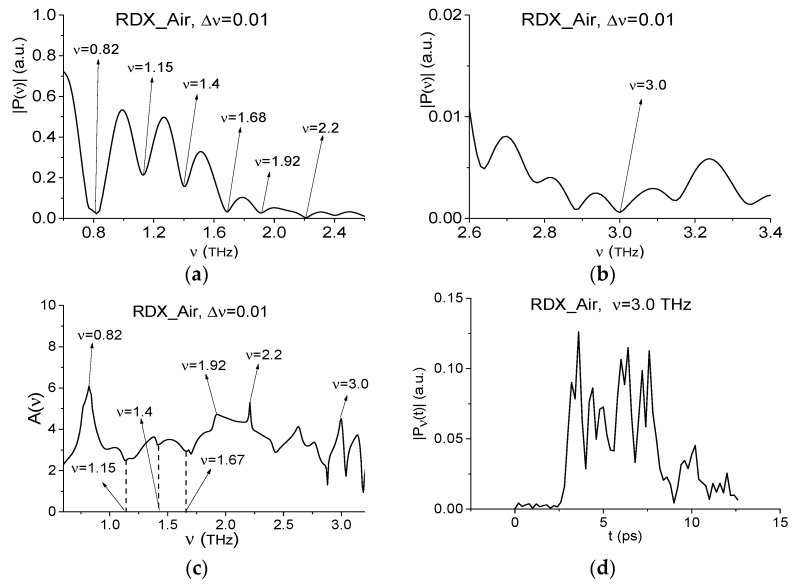
Spectrum (**a**), (**b**) and absorbance (c) of the standard signal RDX_Air depicted in the frequency ranges ν = [0.6, 2.6] THz (**a**), [2.6, 3.4] THz (**b**), and [0.2, 3.2] THz (**c**). Time-dependent evolution of the spectral intensity at the frequency ν = 3.0 THz (**d**).

To find RDX absorption frequencies in the RTH and RTP main pulse spectra, we use the transmitted THz signal RDX_Air (Figure 4) as the standard one and the spectral lines dynamics of this signal at the high frequency ν = 3.0 THz. The RDX_Air signal spectrum is presented in Figure 8 in the frequency ranges ν = [0.6, 2.6] THz (a) and [2.6, 3.4] THz (b). The RDX_Air absorbance is depicted in (c). The spectrum minima at the frequencies ν = 0.82, 1.92, 2.2, 3.0 THz in Figure 8a,b and the peaks in (c) are in a good agreement with the absorption frequencies of pure RDX given in [4,5,57]. In [44] we showed that the spectrum minima at the frequencies ν = 1.15, 1.4, 1.67 THz do not correspond to the absorption frequencies of a pure RDX in our case. They are caused by water vapor containing in the air and cannot be used for the detection and identification of substance. These absorption frequencies agree with those of water vapor reported in Reference [10], namely, ν = 1.17, 1.41 and 1.67 THz. Note that water vapor is always present when measurements are provided under real conditions, this situation is typical for the security screening. In this case using the THz signal, which was measured also under real conditions as a standard one, we exclude the absorption frequencies of water vapor from our consideration. However, such standard THz signals as RDX_Air signal contain the absorption frequencies, which are not disturbed by the water absorption and can be used for the identification of substance. As an example, the absorption frequency ν = 3.0 THz is presented in Figure 8b and we depict the spectral line dynamics at this frequency in Figure 8d. The computation is made with the spectral resolution Δν = 0.01 THz.

**Figure 9:**
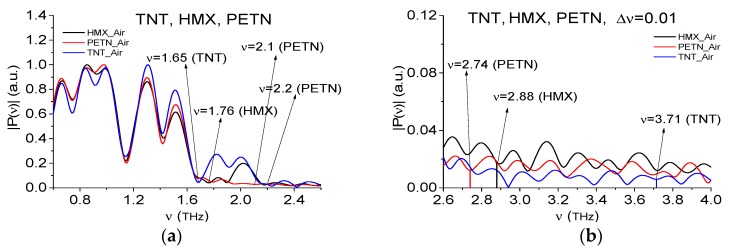

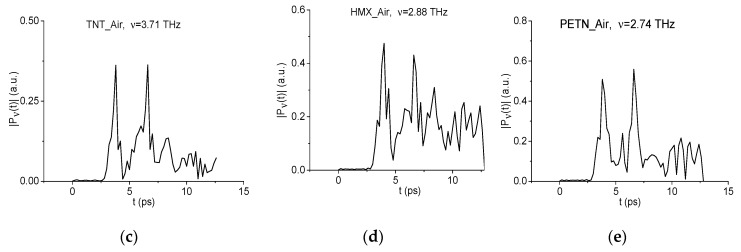
Spectra of the standard signals HMX_Air, PETN_Air and TNT_Air depicted in the frequency ranges ν = [0.6, 2.6] THz (**a**) and [2.6, 4.0] THz (**b**). Spectral line dynamics at the frequencies ν = 3.71 THz (TNT_Air) (**c**), 2.88 THz (HMX_Air) (d), and 2.74 THz (PETN_Air) (**e**).

For the detection of the TNT, HMX, and PETN absorption frequencies in the RTH and RTP main pulse spectra, we use the standard THz signals TNT_Air, HMX_Air, and PETN_Air (Figure 4), which spectra are shown in Figure 9 in the frequency ranges ν = [0.6, 2.6] THz (a) and [2.6, 4.0] THz (b). They are also computed with the spectral resolution Δν = 0.01 THz. For substance detection, we use the frequency ν = 3.68 THz in the RTH main pulse spectrum and the frequency ν = 3.72 THz in that of RTP signal, depicted in Figure 2. Both frequencies are close to the known TNT absorption frequency ν = 3.69 THz [5] and to the absorption frequency ν = 3.71 THz depicted in Figure 9b. The spectral line dynamics at the frequency ν = 3.71 is shown in Figure 9c. Other absorption frequencies ν = 2.88 THz and ν = 2.74 THz agree with those given in References [4,5] for HMX and PETN, respectively. We notice that some discrepancies between the corresponding values can be caused by the different sample preparation (tablet thickness, weight, density). The standard spectral lines dynamics computed at these frequencies are shown in Figure 9d,e.

**Figure 10:**
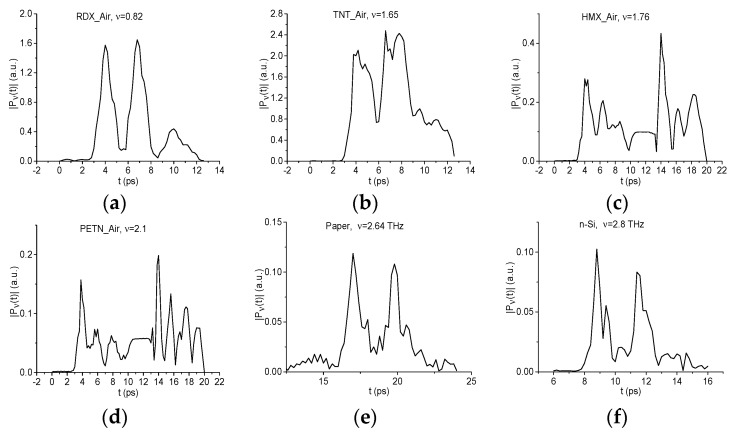
Spectral line dynamics at the frequencies ν = 0.82 THz (RDX_Air) (**a**), 1.65 THz (TNT_Air) (**b**), 1.76 THz (HMX_Air) (**c**), 2.1 THz (PETN_Air) (**d**), 2.64 THz (paper) (**e**), and 2.8 THz (n-Si) (**f**).

Since below other parts of the measured signals are used for the substance detection, the spectral line dynamics at the frequencies ν = 0.82 THz (RDX_Air), 1.65 THz (TNT_Air), 1.76 THz (HMX_Air), 2.1 THz (PETN_Air), 2.64 THz (paper), and 2.8 THz (n-Si) are shown in Figure 10a–f.

### 2.5. Similarity of the Spectral Line Dynamics Shape for the Standard Signals

**Figure 11:**
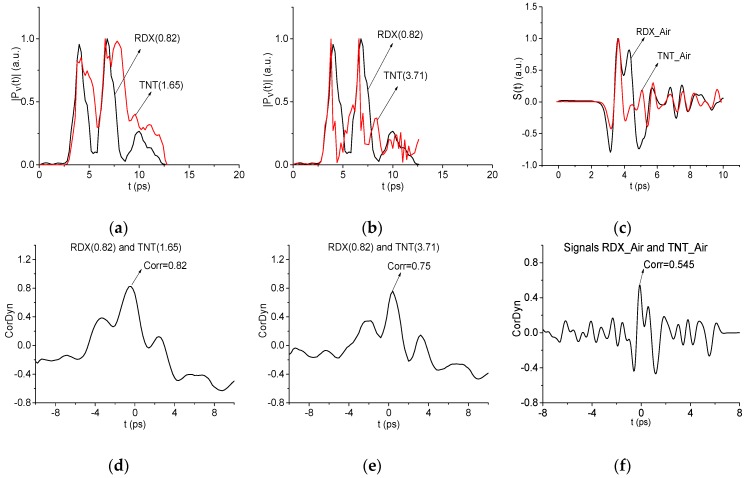
Normalized spectral line dynamics RDX (0.82), TNT(1.65) (**a**), RDX (0.82), TNT(3.71) (**b**); standard THz signals RDX_Air and TNT_Air (**c**); correlation coefficient between spectral line dynamics RDX(0.82) and TNT(1.65) (**d**); RDX(0.82), and TNT(3.71) (**e**); between standard signals RDX_Air and TNT_Air (**f**).

Spectral line dynamics of different standard substances may turn out to be similar because the corresponding relaxation times of excited energy levels may be equal. Accordingly, the question arises about the unambiguity of the identification using similar time-dependent spectral line dynamics. To answer this question, we explore the identification of RDX using the absorption frequency ν = 0.82 THz in the RTH signal and the same frequency of the standard signal RDX_Air as well as the frequencies ν = 1.65 and 3.71 THz of the standard signal TNT_Air. For brevity, we denote these dynamics as dynamics of RDX(0.82), TNT(1.65), and TNT(3.71). All these frequencies are the minima of the RDX_Air and TNT_Air spectra (Figure 8 and Figure 9). In Figure 11 the normalized in C-norm spectral line dynamics RDX(0.82), TNT(1.65) (a) and RDX(0.82), TNT(3.71) (b) are depicted. Moving the spectral line dynamics TNT(1.65) and TNT(3.71) along the spectral line dynamics RDX(0.82), we obtain the correlation coefficient $c_{p,P}(t_{n})$ (1) between them with maximal values: $c_{TNT(1.65),RDX(0.82)}$ = 0.82 (d) and $c_{TNT(3.71),RDX(0.82)}$ = 0.75 (e). It is worth noting that despite the rather high correlation between the spectral line dynamics, their shapes have obvious differences, which results in different values of the corresponding ICCs $C_{p,P}$ (2), $CW1_{p,P}$ (4) and detection of the RDX absorption frequency ν = 0.82 THz in the RTH signal. In turn, we see in (c) that the correlation coefficient $c_{TNT,RDX}$ between the standard signals RDX_Air and TNT_Air, normalized in C-norm and depicted in (f), achieves a maximal value $c_{TNT,RDX}$ = 0.545. It means that the shapes of the standard signals in the time domain are quite different, and this yields the differences in the frequency domain. The negative values of the correlation coefficient in (d) and (e) for |t| > 4 ps, is due to the averaging of the spectral line dynamics in order to avoid the influence of the constant component on the correlation, see (1).

In Section 2.9, we will show that despite the high correlation between the spectral line dynamics TNT (1.65), TNT (3.71), and RDX (0.82) the use of ICCs does not lead to the false detection of the substance that is not present in the mixture.

### 2.6. Efficiency of Substance Detection in the Time Interval t = [0, 25] ps, Containing the Main Reflected Pulse

We see the absence of pronounced minima in the main pulse spectra in the low frequency range (ν < 2.6 THz), Figure 2a. Nevertheless, there are the spectrum minima in the high frequency range (ν > 2.6 THz), which may correspond to the absorption frequencies of the mixtures components and can be used for the substance detection and identification.

**Figure 12:**
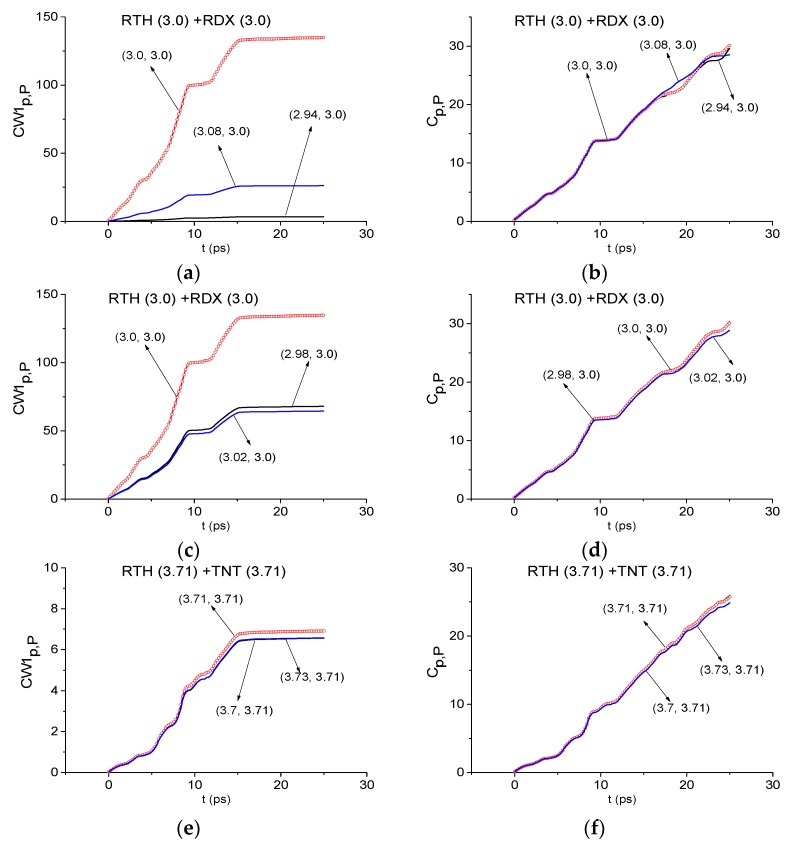
Time-dependent ICC $CW1_{p,P}$ (a,c,e) and $C_{p,P}$ (**b,d,f**) computed at the frequencies ν = 3.0 THz (**a–d**), 3.71 THz (**e,f**) using the FDRs *ν* = [2.94, 3.08] THz (**a,b**), [2.98, 3.02] THz (**c,d**), and [3.7, 3.73] THz (**e,f**).

First, we will detect the absorption frequencies of the common components of two mixtures – RDX and TNT. In Figure 12 the evolution of the ICC $CW1_{p,P}$ (a), (c) and $C_{p,P}$ (b), (d), is shown for the frequency *ν* = 3.0 THz in the decreasing FDRs *ν* = [2.94, 3.08] THz (a), (b) and [2.98, 3.02] THz (c), (d). Note, if the boundaries of FDR in (a), (b) are chosen to be the spectral maxima closest to the standard signal RDX_Air spectrum minimum at the frequency *ν* = 3.0 THz (Figure 8b), then only the ICC $CW1_{p,P}$ detects this frequency as the RDX absorption frequency in the time interval t = [0, 25] ps. The frequency detection based on the ICC $C_{p,P}$ is observed in (b) only in the part of this time interval (t = [0, 17] ps). Probably, this is not enough to take a decision about the detection of this frequency as the RDX absorption frequency. If we decrease the FDR to the range, in which both ICCs detect the desired frequency in the full time interval simultaneously, then this frequency is the RDX absorption frequency. This aim is reached if we decrease the FDR to the interval *ν* = [2.98, 3.02] THz. We will call this FDR as a minimal FDR. Taking into account that the reference spectrum minimum at the frequency *ν* = 3.0 THz is absent (Figure 2), we conclude that this spectral minimum in RTH main pulse spectrum does not appear due to the noise or environment influence. Therefore, the frequency *ν* = 3.0 THz is the RDX absorption frequency. The similar result occurs for the main RTP pulse with the same minimal FDR.

In Figure 12e,f the ICC $CW1_{p,P}$ and $C_{p,P}$ are computed for the absorption frequency *ν* = 3.71 THz of the standard TNT_Air signal. In these cases the lines corresponding to the frequency *ν* = 3.71 THz, lie above other lines in the minimal FDR *ν* = [3.7, 3.73] THz. Thus, both ICCs show that this frequency of the RTH main pulse spectrum is the TNT absorption frequency.

The same results occur at the detection of the frequencies *ν* = 2.88 THz, 2.74 THz as the absorption frequencies of HMX/PETN in the RTH/RTP main pulse spectra. In this case, the minimal FDRs are *ν* = [2.87, 2.92] THz and [2.72, 2.75] THz, respectively.

Thus, the presence of the absorption frequencies of the substances RDX, TNT, HMX, and PETN in the RTH and RTP main pulse spectra is demonstrated using the ICCs. All detected absorption frequencies lie in the high frequency range *ν* > 2.6 THz.

### 2.7. The Substance Detection Using the First Sub-Pulse (Time Interval t = [30, 70 ps)

For the substance detection it is very effective to divide the time interval *t* = [25, 180] ps into several time intervals containing the first sub-pulse and the sub-pulses after the first one. The absorption (or emission) at some frequencies may appear after the end of main pulse due to different characteristic times of the molecule energy level relaxation. Since the spectral intensity at these frequencies is significantly lower than the corresponding spectral intensity for the main pulse, they can be easily missed when analyzing the spectrum during the total time interval. At the same time, in the separated time intervals the absorption frequencies with lower spectral intensities can be more clearly visible and therefore may be detected. Let us remember that, despite lower intensities at high absorption frequencies, the inhomogeneous surface or non-opaque covering less destroy them [53,58]. Therefore, they can be used for the detection and identification, especially under real conditions. This increases the reliability of the identification.

**Figure 13:**
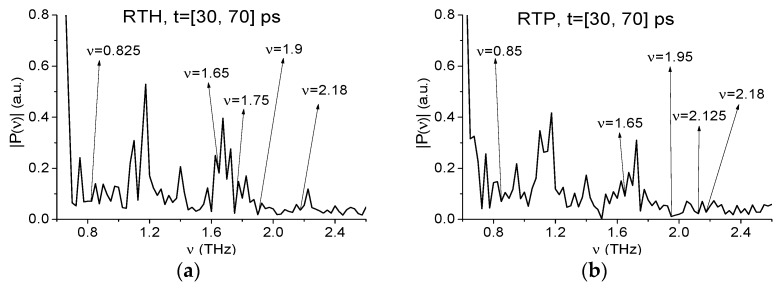

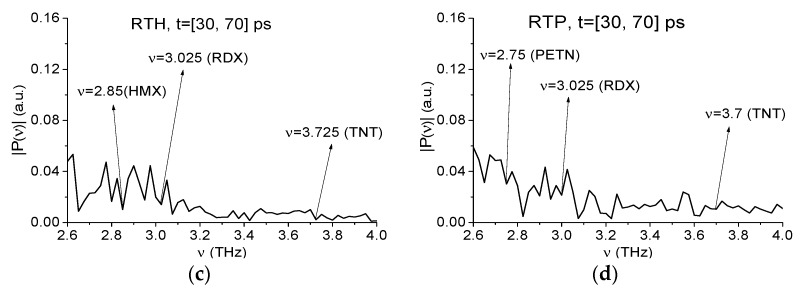
Spectra of the RTH (**a,c**) and RTP (**b,d**) signals computed in the time interval t = [30, 70] ps, in the frequency ranges ν = [0.6, 2.6] THz (**a,b**), and [2.6, 4.0] THz (**c,d**).

We will analyze RTH and RTP signals separately both in the low (ν < 2.6 THz) and high (ν > 2.6 THz) frequency ranges. Earlier in [41] these mixture components were detected and identified by means of the ICC $CW_{p,P}(t_{n})$ (3) in the low frequency range ν < 2.6 THz only. As we discussed above, under real conditions the exploration of high absorption frequencies is preferable. Therefore, in Figure 13 the spectra of the RTH (a), (c) and RTP (b), (d) signals are shown in the frequency ranges ν = [0.6, 2.6] THz (a), (b) and [2.6, 4.0] THz (c), (d) computed at the frequency resolution Δν = 0.025 THz. In Figure 13a,b one can see the spectrum minima at the frequencies ν = 0.825 THz (a), 0.85 THz (b) close to the known RDX absorption frequency ν = 0.82 THz, and the minima at the frequencies ν = 1.9, 2.18 THz (a) and 1.95, 2.18 THz (b), which are close to the RDX absorption frequencies ν = 1.95, 2.2 THz. As well in (a), (b) there is the spectrum minimum at the frequency ν = 1.65 THz (the corresponding TNT absorption frequency is equal to ν = 1.65 THz). We see also in the RTH spectrum (a) a minimum at the frequency ν = 1.75 THz (close to the HMX absorption frequency ν = 1.76 THz), and in RTP spectrum (b) – at the frequency ν = 2.125 THz (close to the PETN absorption frequency ν = 2.1 THz). In the high frequency range (ν > 2.6 THz) one can observe the spectrum minima at the frequency ν = 3.025 THz close to that of RDX ν = 3.0 THz (Figure 8) in both spectra (Figure 13c,d). In (c) there is a spectrum minimum at the frequency ν = 3.725 THz, and in (d)—a minimum at the frequency ν = 3.7 THz, which are close to that of TNT ν = 3.71 THz.

We stress that belonging of the corresponding absorption frequencies to the mentioned explosives is also confirmed by the high correlation between the standard RDX_Air, TNT_Air, HMX_Air THz signals and the RTH signal (as well as between RDX_Air, TNT_Air, PETN_Air, and RTP signals) during the time interval containing the first sub-pulse, see Figure 5b.

#### 2.7.1. Detection of the Mixture Components in the Low Frequency Range ν < 2.6 THz.

**Figure 14:**
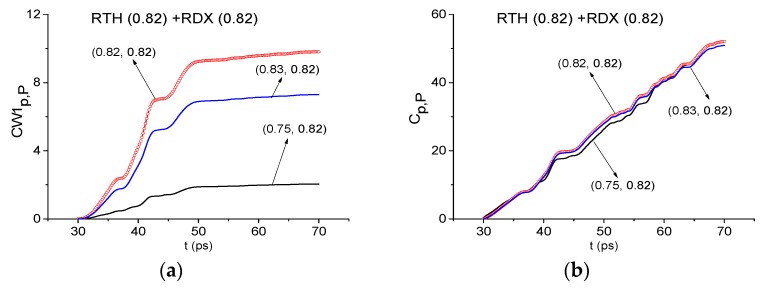
Time-dependent ICCs $CW1_{p,P}$ (a) and $C_{p,P}$ (**b**) computed for the RTH frequency ν = 0.82 THz in the FDR *ν* = [0.75, 0.83] THz.

Firstly, we apply the ICCs to detect the absorption frequencies belonging to the low frequency range ν < 2.6 THz. In Figure 14, the evolution of the ICCs $CW1_{p,P}$ (a) and $C_{p,P}$ (b) are shown for the RTH frequency *ν* = 0.82 THz in the minimal FDR *ν* = [0.75, 0.83] THz. We see that this frequency is detected by both ICCs as the RDX absorption frequency in the full time interval t = [30, 70] ps. If we increase the FDR up to *ν* = [0.75, 0.85] THz or [0.75, 0.84] THz then the detection of the frequency *ν* = 0.82 THz using the ICC $C_{p,P}$ is observed only in the partial time-interval less than t = [30, 70] ps. Essentially, other frequencies *ν* = 1.95, 2.2 THz are also detected in the RTH and RTP signals spectra as the RDX absorption frequencies at using the ICCs $CW1_{p,P}$ and $C_{p,P}$ (not shown). The same result is valid if we analyze the RTP first sub-pulse.

**Figure 15:**
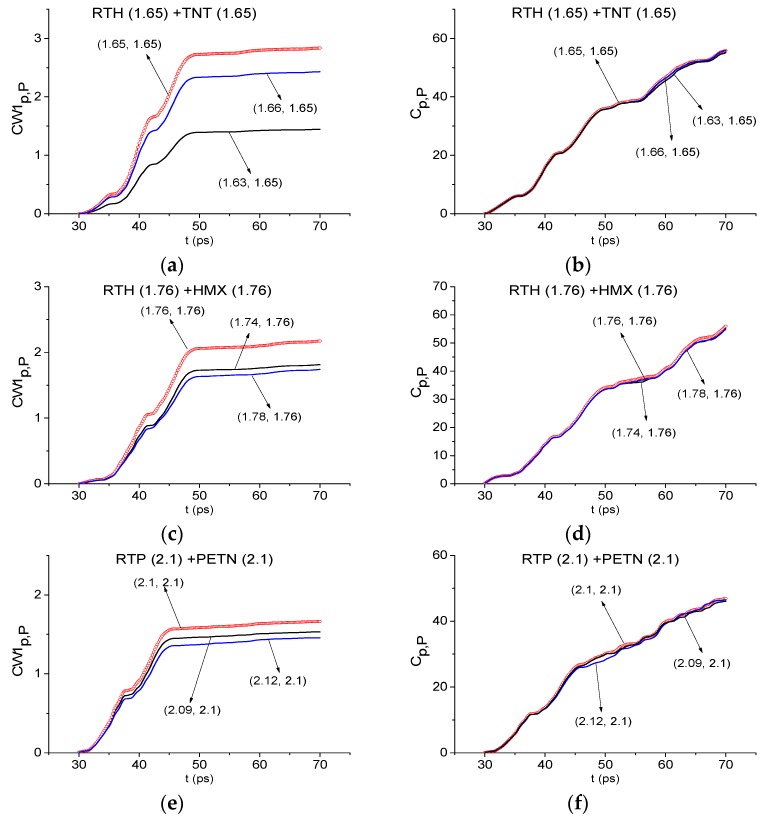
Time-dependent ICCs $CW1_{p,P}$ (**a**,**c**,**e**) and $C_{p,P}$ (**b**,**d**,**f**) detecting the frequencies ν = 1.65 THz (**a**,**b**) and 1.76 THz (**c**,**d**) in the RTH first sub-pulse spectrum, and ν = 2.1 THz (**e**,**f**) in the RTP first sub-pulse spectrum.

Now we discuss the detection of other components of the mixtures. In Figure 15 the ICC $CW1_{p,P}$ and $C_{p,P}$ also detect the frequencies *ν* = 1.65 THz and 1.76 THz as the absorption frequencies of the substances TNT (a), (b) and HMX (c), (d) in the RTH first sub-pulse spectrum. In Figure 15e,f the frequency *ν* = 2.1 THz is detected as the PETN absorption frequency in the RTP first sub-pulse spectrum. In this case, the corresponding minimal FDRs are: *ν* = [1.63, 1.66] THz (a), (b), *ν* = [1.74, 1.78] THz (c), (d) and *ν* = [2.09, 2.12] THz (e), (f). We stress that the frequency *ν* = 1.65 THz is also detected as TNT absorption frequency in the RTP first sub-pulse spectrum with the minimal FDR *ν* = [1.63, 1.66] THz (not shown).

#### 2.7.2. Detection of the Mixture Components in the High Frequency Range ν > 2.6 THz.

**Figure 16:**
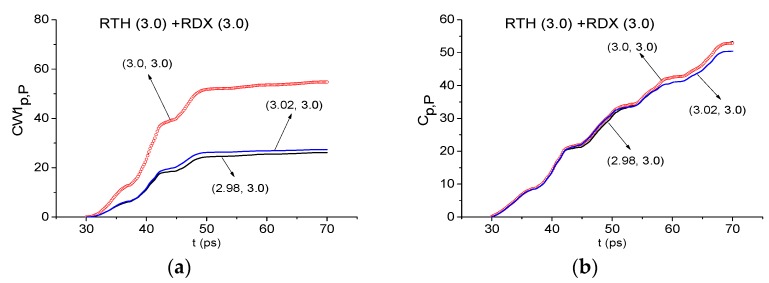

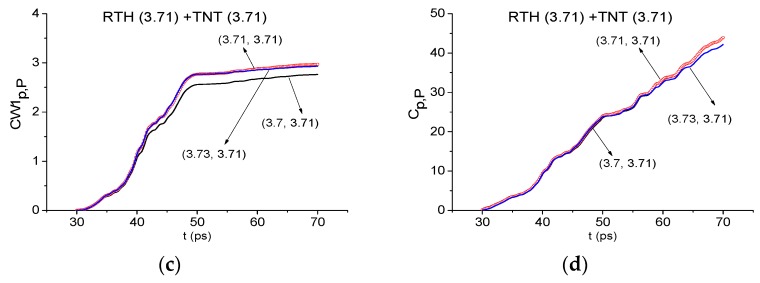
ICCs $CW1_{p,P}$ (a,c), $C_{p,P}$ (**b,d**) detecting the frequencies ν = 3.0 THz (**a,b**) and 3.71 THz (**c,d**) as RDX and TNT absorption frequencies, respectively, in the RTH first sub-pulse spectrum.

Using the ICCs $CW1_{p,P}$ (a), (c) and $C_{p,P}$ (b), (d), one can recognize the frequencies *ν* = 3.0 THz and 3.71 THz as the absorption frequencies of RDX and TNT, respectively, in the RTH first sub-pulse spectrum (Figure 16). The minimal FDRs are *ν* = [2.98, 3.02] THz (a), (b) and [3.7, 3.73] THz (c), (d).

**Figure 17:**
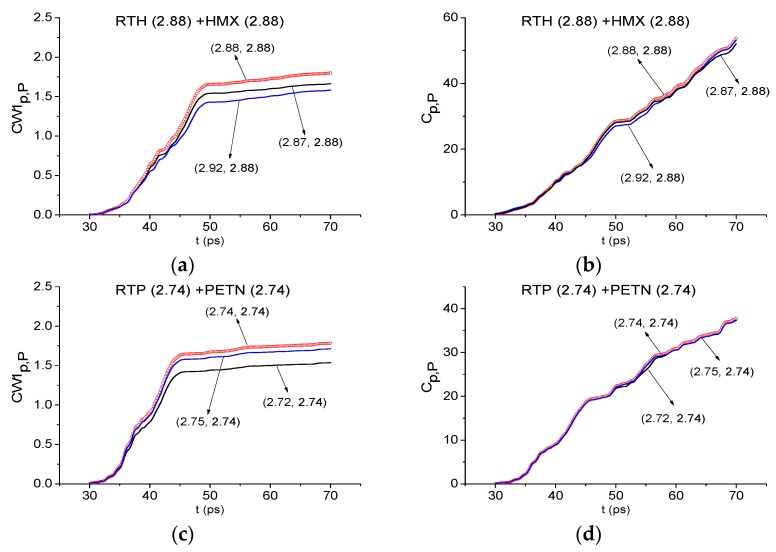
ICCs $CW1_{p,P}$ (a,c) and $C_{p,P}$ (**b,d**) detecting the frequencies ν = 2.88 THz (**a,b**) and 2.74 THz (**c,d**) in the RTH and RTP main pulse spectra.

In Figure 17 the frequencies *ν* = 2.88 THz and 2.74 THz (Figure 8) are detected also as the absorption frequencies of HMX (a), (b) and PETN (c), (d) in the RTH and RTP first sub-pulse spectra. The minimal FDRs are *ν* = [2.87, 2.92] THz (a), (b) and [2.72, 2.75] THz (c), (d), respectively.

Finally, analyzing the first sub-pulse, we see that each of the RTH and RTP mixture components is detected by the corresponding set of the absorption frequencies belonging to the high frequency range *ν >* 2.6 THz, which increases the reliability of the detection and identification of substance. The high correlation between the standard THz signals and the signal under investigation during this time validates a presence of the dangerous substance in the mixture. Obviously, this fact can serve as an additional criterion for the substance detection in both frequency ranges.

Summarizing the results obtained in Section 2.6 and Section 2.7, we conclude that if the standard substance is present in the mixture, then a minimal FDR exists so that both ICCs $CW1_{p,P}$ and $C_{p,P}$ enable to detect simultaneously the frequency corresponding to the spectrum minimum as the absorption frequency of this substance.

### 2.8. The Substance Detection Using the Time Interval t = [70, 170] ps

**Figure 18:**
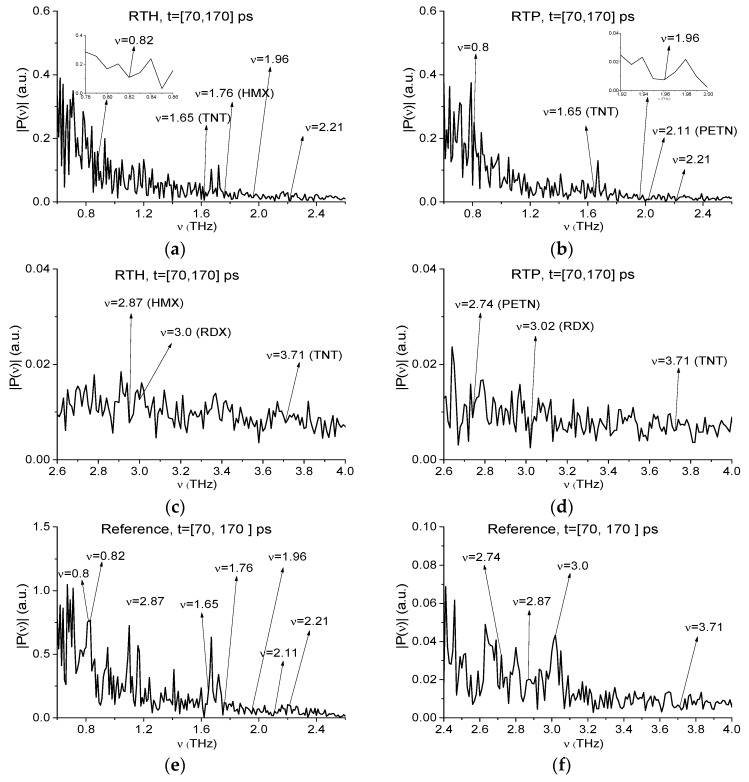
Spectra of the RTH (**a,c**), RTP (**b,d**) signals and reference (**e,f**) computed in the time interval t = [70, 170] ps, in the frequency ranges ν = [0.6, 2.6] THz (**a,b,e**) and [2.6, 4.0] THz (**c,d,f**).

The absorption frequencies of the RTH and RTP mixture components can be detected in the remote time interval *t* = [70, 170] ps, which contains two sub-pulses $S_{2}(t)$ and $S_{3}(t)$. As above, we will consider the low (ν < 2.6 THz) and high (ν > 2.6 THz) frequency ranges separately. With this aim, Figure 18 shows the RTH (a), RTP (b) signals spectra as well as reference spectrum (c) in the frequency ranges ν = [0.6, 2.6] THz (a), (b) and [2.6, 4.0] THz (c), (d) at the spectral resolution ∆ν = 0.01 THz.

#### 2.8.1. Detection of the Mixture Components in The Low Frequency Range ν < 2.6 THz.

In the RTH and RTP spectra (Figure 18) (a), (b)) one can see the minima at the frequencies ν = 0.82 THz (a), 0.8 THz (b) and ν = 1.96, 2.21 THz (a), (b) close to the RDX absorption frequencies ν = 0.82, 1.95, 2.2 THz, respectively (Figure 8). The spectra in (a) and (b) also contain a common minimum at the frequency ν = 1.65 THz (equal to the TNT absorption frequency ν = 1.65 THz). In the RTH spectrum (a) there is a minimum at the frequency ν = 1.76 THz (equal to the HMX absorption frequency ν = 1.76 THz), and in RTP spectrum (b) – at ν = 2.11 THz (close to the PETN absorption frequency ν = 2.1 THz) (Figure 9). In the reference spectrum (c), the minima at these frequencies are absent. It means that these frequencies do not correspond to water vapor.

**Figure 19:**
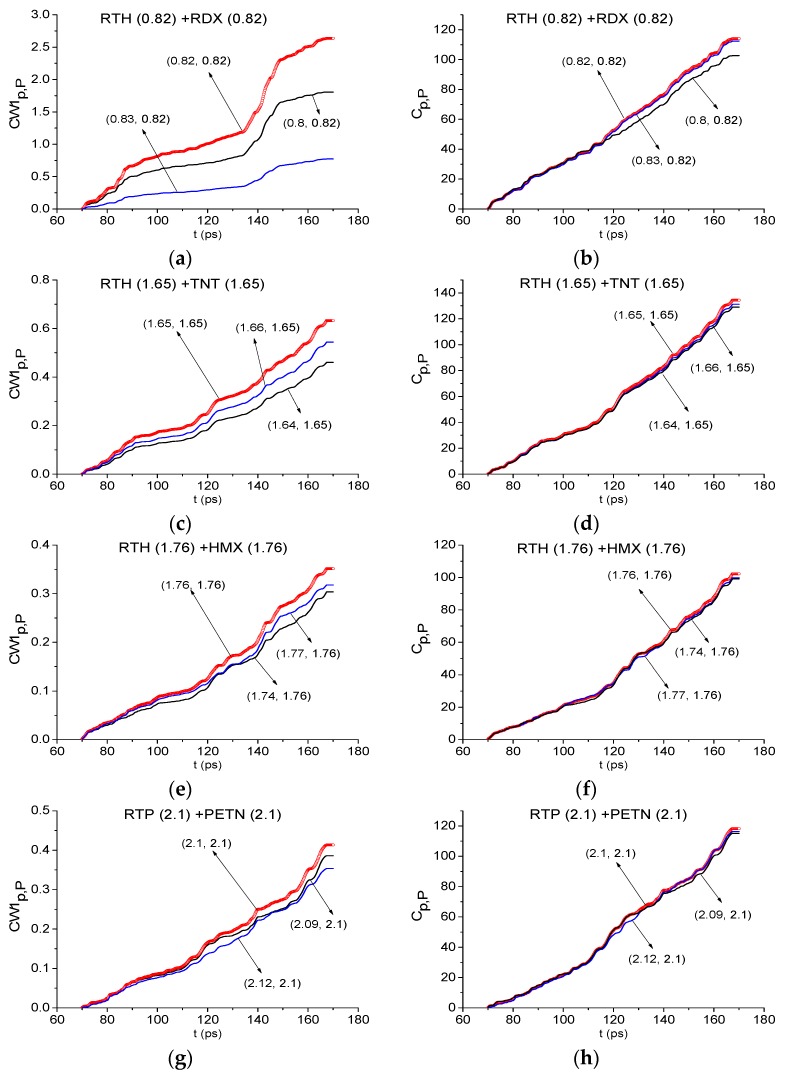
Time-dependent ICCs $CW1_{p,P}$ (**a,c,e,g**) and $C_{p,P}$ (**b,d,f,h**) detecting the frequencies ν = 0.82 THz (RDX) (**a,b**), 1.65 THz (TNT) (**c,d**), 1.76 THz (HMX) (**e,f**) in the RTH signal and ν = 2.1 THz (PETN) (**g,h**) in the RTP signal in the time interval t = [70, 170] ps.

Using the ICCs $CW1_{p,P}$ (a), (c), (e), (g) and $C_{p,P}$ (b), (d), (f), (h) the frequencies ν = 0.82 THz (RDX) (a), (b), 1.65 THz (TNT) (c), (d), 1.76 THz (HMX) (e), (f) are detected in the RTH signal, and the frequency ν = 2.1 THz (g), (h)-in the RTP signal exploring the time interval t = [70, 170] ps (Figure 19). The corresponding minimal FDRs are: *ν* = [0.8, 0.83] THz (RDX) (a) (b), [1.64, 1.66] THz (TNT) (c), (d), [1.74, 1.77] THz (HMX) (e), (f) and [2.09, 2.12] THz (PETN) (g), (h). Note that in the time interval under consideration, the minimal FDRs are less than for the time interval t = [30, 70] ps at exploring the RDX_Air, TNT_Air and HMX_Air standard signals (see Figure 14 and Figure 15). We believe it is because of the lower spectral intensities of the THz signals under investigation and the noise influence.

Let us note that the frequencies ν = 1.96 and 2.2 THz were detected also as the RDX absorption frequencies in the RTH and RTP signals by means of the ICC $CW1_{p,P}$ and $C_{p,P}$ (not shown). The corresponding minimal FDRs are the same as for the RTH signal.

#### 2.8.2. Detection of the Mixture Components in the High Frequency Range ν > 2.6 THz.

To illustrate such possibility, in Figure 18c the spectrum minima at the frequencies ν = 2.87 THz, 3.0 THz and 3.71 THz, which are close the absorption frequencies of HMX, RDX and TNT, respectively (Figure 8 and Figure 9) are depicted. In Figure 18d the spectrum minima at the frequencies ν = 2.74 THz, 3.02 THz and 3.71 THz, which are close the absorption frequencies of PETN, RDX, and TNT, are present. In the reference spectrum (f) the minima at these frequencies are absent.

**Figure 20:**
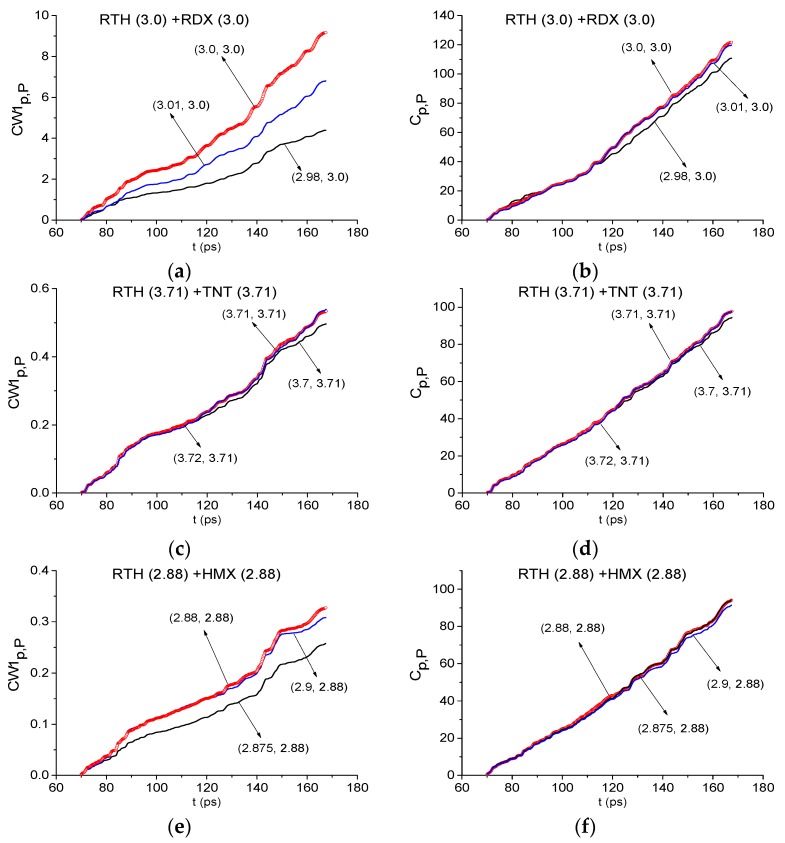

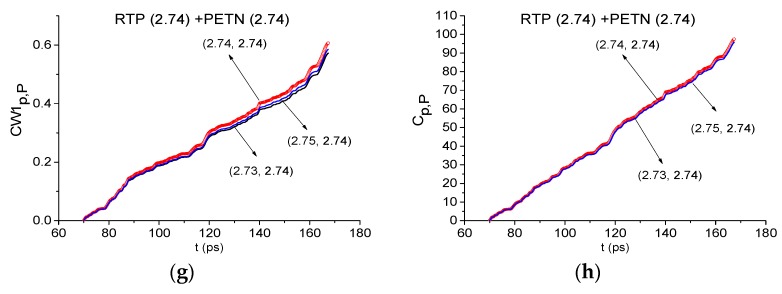
Time-dependent ICC $CW1_{p,P}$ (a,c,e,g) and $C_{p,P}$ (**b,d,f,h**) detecting frequencies ν = 3.0 THz (**a,b**), 3.71 THz (**c,d**), 2.88 THz (**e,f**) and 2.74 THz (**g,h**) in the RTH (**a–f**) and RTP (**g,h**) signals in the time interval t = [70, 170] ps.

In Figure 20 the ICCs $CW1_{p,P}$ and $C_{p,P}$ evolution is depicted at the frequencies ν = 3.0 THz (RDX)(a), (b), 3.71 THz (TNT) (c), (d) and 2.88 THz (HMX) (e), (f) for RTH signal and at the frequency ν = 2.74 THz (PETN) (g), (h) for RTP signal. The corresponding minimal FDRs are: *ν* = [2.98, 3.01] THz (a) (b), [3.7, 3.72] THz (c), (d), [2.875, 2.9] THz (e), (f) and [2.73, 2.75] THz (g), (h).

We see that the ICC $CW1_{p,P}$ detects the frequencies ν = 3.0 THz (a), 3.71 THz (c) and ν = 2.88 THz (e) as the absorption frequencies of RDX, TNT and HMX, respectively, in the RTH signal in the time interval t = [70, 170] ps. The frequency ν = 2.74 THz (g) is detected as the PETN absorption frequency in the RTP signal. The evolution of the ICC $C_{p,P}$ in time at these frequencies confirms a conclusion made above. Note that the frequencies ν = 3.02 THz and 3.71 THz were also detected as RDX and TNT absorption frequencies in the RTP signal by means of the ICCs $CW1_{p,P}$ and $C_{p,P}$.

### 2.9. Can Criteria Distinguish the Frequencies Possessing the Similar Time-Dependent Spectral Line Dynamics?

**Figure 21:**
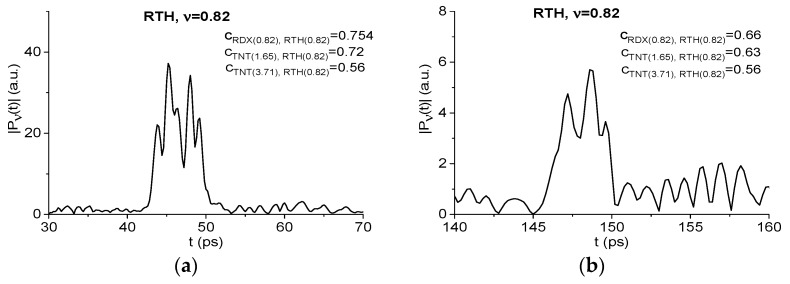
RTH spectral line dynamics computed at the frequency ν = 0.82 THz in the time intervals t = [30, 70] ps (**a**) and [140, 160 ] ps (**b**).

To answer this question, we consider the time intervals t = [30, 70] ps and [140, 160] ps, which contain the first and the third sub-pulses of the RTH signal. The corresponding spectral line dynamics under consideration is shown in Figure 21a,b. Moving the spectral line dynamics RDX(0.82), TNT(1.65), and TNT(3.71) (Figure 11) along the RTH spectral line dynamics at the frequency ν = 0.82 THz (RTH(0.82) for brevity), we obtain the following maximal correlation coefficients in the time interval containing the first sub-pulse *S_1_*(*t*) (a): $c_{RDX(0.82),RTH(0.82)}$ = 0.754, $c_{TNT(1.65),RTH(0.82)}$ = 0.72 and $c_{TNT(3.71),RTH(0.82)}$ = 0.56. For the third sub-pulse *S_3_*(*t*) (b) we compute the following values: $c_{RDX(0.82),RTH(0.82)}$ = 0.66, $c_{TNT(1.65),RTH(0.82)}$ = 0.63 and $c_{TNT(3.71),RTH(0.82)}$ = 0.56. Thus, the maximal correlation is observed between the dynamics RTH(0.82) and RDX(0.82) in both time intervals. It means that the time-dependent evolution of the spectral intensities at these frequencies differs from each other. However, the difference is not pronounced. Does the integral correlation criteria reflect this difference or not?

**Figure 22:**
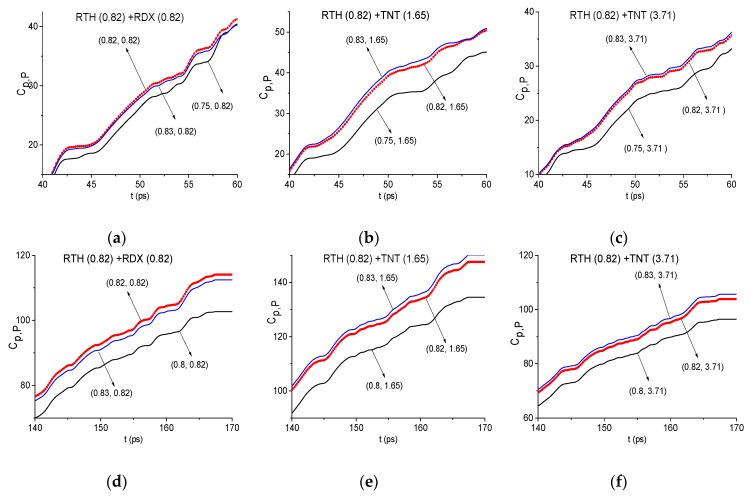
Time-dependent ICC $C_{p,P}$ computed for the dynamics RTH(0.82) and the standard dynamics RDX(0.82) (**a,d**), TNT(1.65) (**b,e**), and TNT(3.71) (**c,f**) in the time intervals t = [40, 60] ps (**a–c**) and [140, 170] ps (**d–f**).

With this aim, in Figure 22 the ICCs $C_{p,P}$ evolution is depicted for RTH(0.82) and RDX(0.82) (a), (d), TNT(1.65) (b), (e) and TNT(3.71) (c), (f) in the time intervals containing the RTH first sub-pulse *S_1_*(*t*) (a)–(c) and the third sub-pulse *S_3_*(*t*) (d)–(f). In (a)–(c) the magnified view is presented in the decreased time interval t = [40, 60] ps and in (d)–(f) - in the time interval t = [140, 170] ps. The corresponding minimal FDRs are *ν* = [0.75, 0.83] THz (a)–(c) and [0.8, 0.83] THz (d)–(f). Obviously, in (a), (d) the evolution of the ICC $C_{p,P}$ corresponding to the standard dynamics RDX (0.82) is the topmost. In (b), (c), (e), and (f), the evolution of the ICC $C_{p,P}$ corresponding to the spectral line dynamics TNT (1.65) and TNT (3.71) instead of RDX (0.82), does not yield the topmost position in the same time intervals as in (a), (d). It should be stressed that without averaging in (1) the corresponding evolutions of the ICC $C_{p,P}$ in (a)–(f) practically coincide, which significantly decreases the effectiveness of the detection.

Thus, the use of the standard dynamics TNT (1.65) and TNT (3.71) instead of RDX (0.82) does not lead to the detection of the absorption frequency *ν* = 0.82 THz in the RTH signal. We see that despite the high correlation between the dynamics TNT (1.65), TNT (3.71) and RDX (0.82), they are not identical, and the differences between them are enough to uniquely identify the substance RDX using the spectral dynamics RDX (0.82).

### 2.10. How to Distinguish the Mixtures RTH and RTP and to Exclude The False Presence Of Paper and Semiconductors in Them?

In the context of a security problem, it is enough to get information about the presence of any dangerous substance at least. However, in practice the detection of all mixture components is an urgent problem. In Figure 2a we see that the RTH and RTP main pulses have similar spectra. It means that the standard THz-TDS method does not allow us to find the pronounced difference between them. Below, we analyze the RTH and RTP signals spectra and reference spectrum simultaneously in the time interval t = [30, 70] ps containing the first sub-pulses. Our purpose is to demonstrate the difference between the absorption frequencies of the mixtures RTH and RTP as well as to exclude the presence of neutral substances paper and Si-based semiconductors within them. The detection of the neutral substances is important for the security screening under real world conditions, because it allows the reduction of false alarms.

The algorithm for the detection of the absence of the substance in the mixture consists of several steps. The first step is to determine the absorption frequencies of the standard substance from the database, for which there are no corresponding minima in the RTH/RTP spectrum. At the second step, we check the presence of minima in the RTH/RTP spectrum that coincide or are close to the absorption frequencies of the standard substance. Then we analyze the reference spectrum in order to check the presence of the same minima in it. If they are present in both spectra, it means that they are caused by water vapor or some environmental influence. If not, we perform the third step and apply the ICC $C_{p,P}$ to show that this minimum in the RTH/RTP spectrum does not belong to the absorption frequencies of the substance of interest. We illustrate this below.

**Figure 23:**
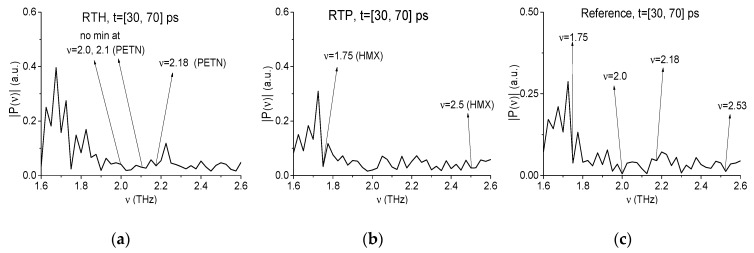
Spectra of the RTH (**a**), RTP (**b**) signals and reference (**c**) computed in the time interval t = [30, 70] ps, in the frequency range ν = [1.6, 2.6] THz.

First, we show the absence of the PETN absorption frequencies in RTH signal spectrum and those of HMX in RTP signal spectrum in this time interval. With this aim, in Figure 23 the spectra of the RTH (a), RTP (b) signals and reference (c) are depicted in the frequency range *ν* = [1.6, 2.6] THz. In the RTH spectrum (a) one can see a minimum at the frequency *ν* = 2.18 THz, which is close to the PETN absorption frequency *ν* = 2.1 THz (Figure 9). The minima at the PETN absorption frequencies *ν* = 2.0, 2.1 THz are absent in (a). In the RTP spectrum (b) there are two minima, which are close to the HMX absorption frequencies, they are *ν* = 1.75, 2.5 THz. At the same time, in the reference spectrum (c) one can see the minima at the close frequencies ν = 2.0, 2.18 THz (for RTH spectrum) and ν = 1.75, 2.53 THz (for RTP spectrum). This means that the corresponding minima in (a) and (b) can also be caused by environment gases.

**Figure 24:**
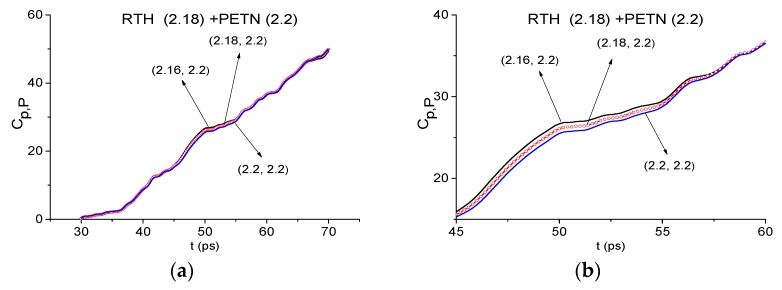
ICC $C_{p,P}$ computed for the frequency ν = 2.18 THz with PETN_Air standard signal in the time intervals t = [30, 70] ps (**a**) and [45, 60] ps (**b**).

The absence of the PETN absorption frequency in the RTH first sub-pulse, for example, *ν* = 2.18 THz, close to the PETN absorption frequency *ν* = 2.16 THz [4,5], can also be confirmed by using the ICC $C_{p,P}$. For this purpose, we use the spectral line dynamics of the standard signal PETN_Air at the frequency *ν* = 2.2 THz (Figure 9a). In Figure 24, this ICC evolution is shown in the FDR *ν* = [2.16, 2.2] THz in the time interval t = [30, 70] ps (a) and the frequency *ν* = 2.18 THz is not recognized as the PETN absorption frequency in the RTH first sub-pulse spectrum. In the shortened time interval t = [45, 60] ps (b), it is clearly seen that the line corresponding to the frequency *ν* = 2.18 THz is not the topmost. The absence of HMX absorption frequencies *ν* = 1.75 THz and 2.5 THz in the RTH first sub-pulse spectrum can be shown in the same way.

**Figure 25:**
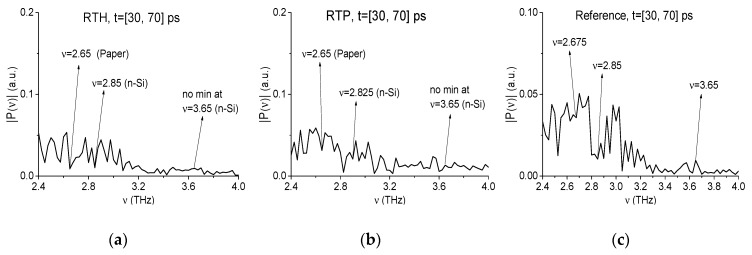
Spectra of the RTH (**a**), RTP (**b**) signals and reference (**c**) computed in the time interval t = [30, 70] ps, in the frequency range ν = [2.4, 4.0] THz.

In the same manner, we show the absence of the paper and n-Si absorption frequencies in the RTH and RTP first sub-pulses spectra. To provide this, the spectra of the RTH (a), RTP (b) signals and reference (c) are depicted in Figure 25 in the frequency range *ν* = [2.4, 4.0] THz. In the RTH and RTP spectra (a), (b) there is a minimum at the frequency *ν* = 2.65 THz (a), (b), which is close to the absorption frequency *ν* = 2.64 THz of the signal paper (see Figure 3). Two minima at the frequencies *ν* = 2.85 THz (a) and 2.825 THz (b) are close to the absorption frequency *ν* = 2.8 THz (Figure 3) of the n-Si signal. In the reference spectrum (c) one can see the minima at the close frequencies ν = 2.675 THz and 2.85 THz. Thus, the corresponding minima in (a) and (b) can also be caused by the same effects as in the reference spectrum. At the same time, the spectrum minimum at the frequency ν = 3.65 THz (close the n-Si absorption frequency ν = 3.64 THz) is absent in the RTH and RTP spectra.

**Figure 26:**
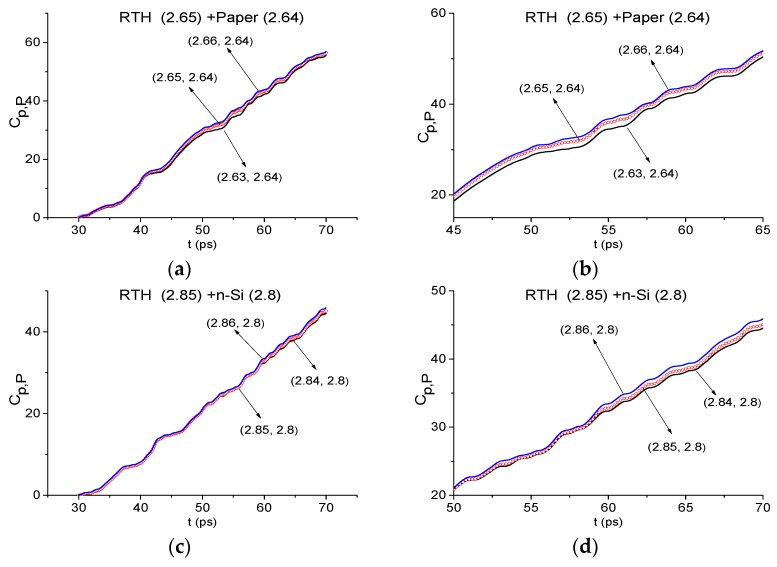
ICC $C_{p,P}$ computed for the frequencies ν = 2.65 THz (**a,b**) and 2.85 THz (**c,d**) in the time intervals t = [30, 70] ps (**a,c**), [45, 65] ps (**b**) and [50, 70] (**d**) with paper (**a,b**) and n-Si (**c,d**) standard signals.

The absence of paper and n-Si absorption frequencies *ν* = 2.65 THz and *ν* = 2.85 THz in the RTH first sub-pulse spectrum can be done as above, by using the ICC $C_{p,P}$. For this purpose, we will use the spectral line dynamics of the standard signals paper and n-Si at the corresponding frequencies *ν* = 2.64 THz and 2.8 THz, which are depicted in Figure 10. In Figure 26 the ICC $C_{p,P}$ is shown in the time interval t = [30, 70] ps (a), (c). This criterion shows that the paper absorption frequency *ν* = 2.65 THz and n-Si absorption frequency *ν* = 2.85 THz are absent in the RTH first sub-pulse spectrum. In (b), (d) the magnified view of Figure 26a,c, respectively, is presented, where we can see that these spectral lines corresponding to the RTH frequencies *ν* = 2.65 THz (b) and 2.85 THz (d) are not the topmost. We note that the corresponding minimal FDRs are *ν* = [2.63, 2.66] THz (a), (b) and [2.84, 2.86] THz (c), (d).

The absence of the absorption frequencies *ν* = 2.65 THz and *ν* = 2.825 THz, corresponding to paper and n-Si samples, in the RTP first sub-pulse spectrum can be achieved in the same way by using the ICC $C_{p,P}$. As well, the absence of absorption frequencies of amphetamine-type illicit drugs MA, MDA, MDMA in the RTH and RTP first sub-pulse spectra can be shown in the same manner [45]. A similar result is achieved in the time interval t = [70,170] ps by using the ICC $C_{p,P}$ and analysis of the reference spectrum (not shown).

Thus, using the ICC $C_{p,P}$ together with analysis of the reference spectrum allows us to distinguish the mixtures RTH and RTP with similar absorption frequencies as well to show the absence of substances of interest in them. This result is extremely important for security screening under real conditions because it allows decreasing h false alarms

## 3. Conclusions and Summarizing

An effective method of pulsed THz spectroscopy is proposed for the detection and identification of ternary mixtures RDX-TNT-HMX (RTH) and RDX-TNT-PETN (RTP) using a long-duration THz signal reflected from the sample. The method discussed does not require a large number of measurements and samples (training sets) for developing of database of mixtures containing their components in various proportions. For the detection and identification of substance with high probability, the reflected THz signal must contain at least one of the sub-pulses. If the structure of sub-pulses is absent then it is necessary to analyze the THz radiation following the main pulse.

The mixture component absorption frequencies belonging to low (ν < 2.6 THz) and high (ν > 2.6 THz) frequency ranges are detected at using both the main pulse and sub-pulses. The RTH and RTP main pulses are shown to contain information about the high absorption frequencies (ν > 2.6 THz) of the mixture components. It is also very important for the practical applications of the discussed method, because inhomogeneous surface or non-opaque covering distort the high absorption frequencies spectral dynamics less.

We detect the substance by using the ICCs $CW1_{p,P}$ and $C_{p,P}$ simultaneously over the partial non-overlapping time intervals. The first time-interval contains the main pulse only, the second time interval - the first sub-pulse and the third interval - the following sub-pulses with lower amplitude. Use of different time intervals increases the reliability and probability of the identification.

We stress that the detection of the mixture components by using the sub-pulses with low amplitudes in comparison with the main pulse amplitude, is very important because it demonstrates the possibility of the substance identification at long distance. Indeed, in this case, the amplitude of a noise THz signal will be comparable with the noise amplitude. Therefore, the main pulse and sub-pulses will be hardly distinguished. The SDA-method allows us to determine the location of the main pulse in the noise THz signal and then using of the ICCs allows us to detect the substance of interest. For additional verification of the substance detection, we use the integral correlation $C_{s,S}$ between the reflected THz signal and the standard THz signal from database simultaneously with other criteria. This technique allows us to significantly increase the probability and reliability of the substance detection and identification.

The algorithm of the substance detection in the mixture RTH/RTP consists of several steps. During the first step, we divide the time interval of the THz signal into several parts. Each of these parts contains separate sub-pulses of the signal (the main pulse, the first sub-pulse and the sub-pulses with lower amplitudes). During the second step, we find the minima in the RTH/RTP spectrum, which are equal or close to the known absorption frequencies of the standard substances from the database. We subsequently analyze the reference spectrum in order to verify the presence/absence of these minima. Over the third step, we apply the ICCs $CW1_{p,P}$ and $C_{p,P}$ to show that these minima in the RTH/RTP spectrum belong to the absorption frequencies of the substance of interest. During the fourth step, we estimate the correlation between the RTH/RTP THz signal and the standard signal from the database.

**Figure 27:**
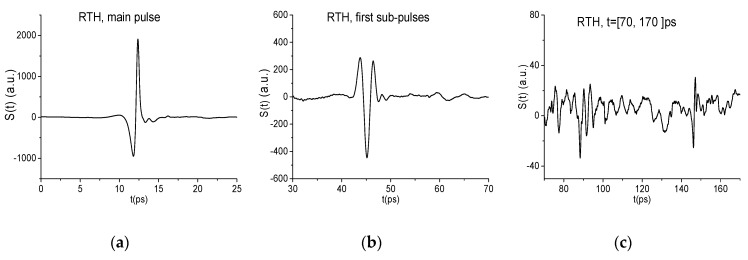

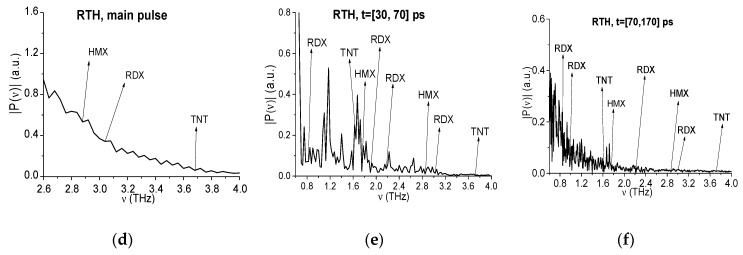
RTH signal in the time intervals t = [0, 25] ps (**a**), [30, 70] ps (**b**), and [70, 170] (**c**). Spectra of the main pulse (**d**), first sub-pulse (**e**) and the remote part of the RTH signal (**f**) with absorption frequencies of RDX, HMX, and TNT detected by the ICCs in the frequency ranges ν = [2.6, 4.0] THz (**d**), [0.6, 4.0] THz (**e,f**).

Summarizing the results obtained in the current paper, in Figure 27 we show the investigated parts of the reflected THz RTH signal (a)–(c) and their spectra with the marked absorption frequencies of the mixture components (e)–(f), which were detected using the ICCs. It is important to note that the selected parts (a)–(c) are the time domains corresponding to the maximum correlation between the standard THz signals and the measured THz signal. They contain the main pulse, the first sub-pulse and the subsequent sub-pulses. In the RTH main pulse spectrum (d) the single absorption frequencies of RDX, HMX and TNT are detected in the high (ν > 2.6 THz) frequency range. Each of the mixture components is detected by the corresponding set of the absorption frequencies in both low (ν < 2.6 THz) and high (ν > 2.6 THz) frequency ranges belonging to the spectra of the first sub-pulse (e) or of the remote part of the RTH signal (f). The similar results are valid for the RTP mixture.

The method discussed in this article allows us not only to detect the presence of hazardous substances in the ternary mixtures, but also to show the absence of the neutral substances, possessing the absorption frequencies similar to those of hazardous substances. For this aim, we use the ICC $C_{p,P}$ together with the analysis of the reference spectrum. This makes it possible to distinguish the mixtures RTH and RTP and to avoid the false alarm at the analysis of the mixtures.

We showed that each of the time-dependent spectral intensities at chosen frequencies, belonging to the standard signals from database, possesses unique property and using a false spectral line dynamics instead of the true spectral dynamics does not lead to the detection of the false absorption frequency.

In conclusion, the SDA-method together with the ICCs provides an effective way for the detection and identification of ternary mixtures in the reflection mode. The discussed ICCs demonstrate both high probability of the substance identification and a reliability of the detection technique in practice. The technique described will be very useful for not only developing the THz security screening devices but also for a quality control in the chemical, biomedical, pharmaceutical, and food industries.
